# Pathogen-triggered metabolic adjustments to potato virus Y infection in potato

**DOI:** 10.3389/fpls.2022.1031629

**Published:** 2023-02-20

**Authors:** Richard Manasseh, Anna Berim, Madhu Kappagantu, Lindani Moyo, David R. Gang, Hanu R. Pappu

**Affiliations:** ^1^ Department of Plant Pathology, Washington State University, Pullman, WA, United States; ^2^ Institute of Biological Chemistry, Washington State University, Pullman, WA, United States

**Keywords:** potato, potato virus Y, metabolomics, plant–virus interaction, gas-chromatography, mass spectrometry

## Abstract

Potato (*Solanum tuberosum L*) is affected by several viral pathogens with the most economically damaging being potato virus Y (PVY). At least nine biologically distinct variants of PVY are known to attack potato, with necrotic types named PVY^NTN^ and PVY^N-Wi^ being the most recent additions to the list. So far, the molecular plant-virus interactions underlying this pathogenicity are not fully understood. In this study, gas chromatography coupled with mass spectroscopy (GC–MS) was used for an untargeted investigation of the changes in leaf metabolomes of PVY-resistant cultivar Premier Russet, and a susceptible cultivar, Russet Burbank, following inoculation with three PVY strains, PVY^NTN^, PVY^N-Wi^, and PVY^O^. Analysis of the resulting GC–MS spectra with the online software Metaboanalyst (version 5.0) uncovered several common and strain-specific metabolites that are induced by PVY inoculation. In Premier Russet, the major overlap in differential accumulation was found between PVY^N-Wi^ and PVY^O^. However, the 14 significant pathways occurred solely due to PVY^N-Wi^. In contrast, the main overlap in differential metabolite profiles and pathways in Russet Burbank was between PVY^NTN^ and PVY^O^. Overall, limited overlap was observed between PVY^NTN^ and PVY^N-Wi^. As a result, PVY^N-Wi^-induced necrosis may be mechanistically distinguishable from that of PVY^NTN^. Furthermore, 10 common and seven cultivar-specific metabolites as potential indicators of PVY infection and susceptibility/resistance were identified by using PLS-DA and ANOVA. In Russet Burbank, glucose-6-phosphate and fructose-6-phosphate were particularly affected by strain–time interaction. This highlights the relevance of the regulation of carbohydrate metabolism for defense against PVY. Some strain- and cultivar-dependent metabolite changes were also observed, reflecting the known genetic resistance–susceptibility dichotomy between the two cultivars. Consequently, engineering broad-spectrum resistance may be the most effective breeding strategy for managing these necrotic strains of PVY.

## Introduction

Plant viruses are among the most important phytopathogens worldwide, with nearly half of the emerging epidemics having viral etiology (Song et al., 2010). It is estimated that virus-induced crop diseases cause economic losses of nearly US$60 billion per year globally. As a result, proper understanding of the mechanisms that contribute to the burden imposed by viral infections has become an important area of research.

Potato (*Solanum tuberosum* L.), the fourth most important food crop in the world (Scholthof et al., 2011), is affected by several important viral pathogens on a worldwide basis (Kreuze et al., 2020), and among them, potato virus Y (PVY, genus *Potyvirus*, family *Potyviridae*) (Lacomme et al., 2017). At least nine biologically distinct strains of PVY have been reported in potato (Karasev and Gray, 2013). These include the relatively new recombinant types named PVY^NTN^ and PVY^N-Wi^, which have almost replaced the traditional strains as the most prevalent in USA. (Dupuis et al., 2019). Both PVY^NTN^ and PVY^N-Wi^ are derived from the same parental strains PVY^N^ and PVY^O^. Whole-genome sequence comparisons of PVY^N^ and PVY^O^ show them to differ by only ≈8% along the entire 9.7-kb genome (Karasev et al., 2011).

In terms of effects on host phenotype, PVY^NTN^ and PVY^N-Wi^, like their progenitors, usually induce only mild or asymptomatic responses on potato foliage (Gray et al., 2010). In contrast, both strains are major triggers of tuber necrosis in susceptible cultivars. Specifically, PVY^N-Wi^ induces tuber cracking, while PVY^NTN^ infections are associated with potato tuber necrotic ringspot disease, which manifests as necrotic ringspots on the tuber surface that extend into flesh (Karasev and Gray, 2013; Benedict et al., 2015).

So far, the molecular plant-virus interactions underlying PVY pathogenicity have been studied mainly by analysis of host gene products, which have revealed PVY-induced alterations involving gene groups and pathways related to photosynthesis, perception, signaling and defense (Baebler et al., 2009; Kogovšek et al., 2010; Baebler et al., 2011; Goyer et al., 2015). On the other hand, the metabolome, which often correlates poorly with gene expression but directly mediates these phenotypic outcomes, is poorly studied.

Given that the delineation of virus–responsive metabolic pathways can pinpoint precise targets that could be exploited for potato improvement through metabolic engineering, this study used gas chromatography-mass spectrometry (GC-MS) to investigate the responses of PVY-resistant cv. Premier Russet and susceptible cv. Russet Burbank to different PVY strains, focusing on untargeted analyses of the leaf metabolome. The study showed that, in general, PVY infection in potato leads to marked changes in amino acid, energy, and fatty acid metabolism. In both cultivars, major overlaps between sets of differential metabolite and pathway alterations induced by the different strains of PVY were observed. In the resistant cultivar, the major overlap occurred between PVY^N-Wi^ and PVY^O^, while in the susceptible cultivar, the main overlap was between PVY^NTN^ and PVY^O^. In addition, a large subset of metabolite and pathway alterations were found to be unique to PVY^N-Wi^-inoculated cv. *Premier Russet*. What causes this is still unclear, but may reflect a larger metabolic energy demand imposed by PVY^N-Wi^ infection, suggesting its necrosis may be mechanistically distinguishable from that of PVY^NTN^.

## Materials and methods

### Experimental design and growth conditions

Potato cultivars *Russet Burbank* and *Premier Russet* were used as the test genotypes. A single potted plant was taken as the experimental unit, with cultivar and PVY strain as the treatment factors. For the PVY strain factor, four levels were included: inoculation with the strains PVY^O^, PVY^NTN^ and PVY^N-Wi^, plus one control inoculation without PVY. Three replications of each treatment (cultivar-strain combination) were included, same as used previously (López-Gresa et al., 2012). Test plants were grown from node tissue culture and maintained in a conditioned greenhouse (soil, 21 ± 2°C, with a photoperiod of 16 hours and relative humidity of 70%). To offset any micro-environment variation within the greenhouse, experimental units were arranged in BugDorm-2400 Insect Rearing Tents according to a Randomized Complete Block Design (RCBD), with a tent as the block. Throughout the experiment, optimal soil moisture was maintained by frequent irrigation.

### Plant inoculations and leaf sampling

Four weeks after transplanting, two fully expanded compound leaves selected from the medium canopy of each potted plant were inoculated as described in Goyer et al. (2015). For virus inoculation, tobacco plants previously infected with PVY^NTN^, PVY^N-Wi^, and PVY^O^ were used as source of inoculum. Strain inocula were prepared by grinding 0.2 g of the infected tobacco leaves in 20 ml of 100 mM sodium phosphate buffer (pH 7.2) containing 0.4% (v/v) β-mercaptoethanol, and 1:10 (wt/vol) of carborundum and celite.

To confirm the PVY status of the inoculum and ensure that inoculations were performed using comparable and effective/infectious doses of PVY, the final inoculum concentrations were standardized using a commercially available double antibody sandwich ELISA (DAS-ELISA) kit (SRA 20001, Agdia, Inc., Elkhart, IN, USA). For this, split samples of the infected tobacco leaf tissues were first ground in general extract buffer (GEB) (Agdia, Inc.). Subsequently, stepwise dilutions (100:0, 100:1, 100:2, 100:4, 100:8, 100:16, and 100:32) of the resulting suspensions were prepared in 100mL of GEB and assayed for antigen levels using DAS-ELISA, with two technical replicates for each sample. A sample was considered positive when the mean absorbance at 405 nm (A405) was higher than twice the value of the buffer (or healthy tissue extract) control, the threshold specified in the manufacturer’s manual. Consequently, diluents with similar A405 above the threshold were used for inoculation.

All the leaflets on each of the three compound leaves were then inoculated by rubbing the inoculum on the adaxial side. For the controls, five plants of each cultivar were mock*-*inoculated with sap of healthy tobacco plants prepared as described above. At seven days post‐inoculation (dpi), two leaflets–one from each inoculated compound leaf were harvested. Two systemically infected leaflets were sampled at 21dpi. Before GC-MS analysis, the infection status of systemic leaf samples was verified by immunostrip testing (Agdia Inc.). Finally, observations of onset and progression of symptoms in the inoculated or younger uninoculated leaves were assessed at weekly intervals starting from the day of inoculation.

### Metabolite extraction

Leaf metabolite extraction was performed using a slight modification of the procedure established by Lee and Fiehn (2008). After the frozen leaf tissues were ground in liquid nitrogen, 100 mg of the powder was suspended in 1000μL of extraction solvent containing methanol, 2-propanol, and water at a ratio of 5:2:2 (v/v). D6-salicylic acid (CDN Isotopes, Quebec, Canada) was then added (1.5μg) as internal standard, and the material vortex-mixed at room temperature for 10 minutes. After vortexing, the suspensions were sonicated at room temperature for 10 minutes using a Branson 5510 ultrasonic sonication bath (Branson Ultrasonics, Danbury, CT, USA). To pellet debris, the samples were then centrifuged for 10 minutes at 21,000 x g. After centrifugation, 200μL of the resulting supernatants (metabolite phase) were then decanted into new vials and evaporated to dryness under vacuum. Dry residues were each reconstituted in 200μL of 50% aqueous acetonitrile and re-extracted by sequential vortexing and sonication as described above. Debris was again pelleted by centrifugation, and the supernatants dried under vacuum.

### Sample derivatization

Prior to GC-MS analysis, the dry residues were derivatized in two steps. For the methoximation step, the dried aqueous phase samples were suspended in 10μL of 30mg/mL O-methoxylamine hydrochloride dissolved in pyridine (Sigma-Aldrich, St. Louis, MO). After vortexing for 5 minutes, the samples were then incubated on an Eppendorf Thermomixer at 30°C, 1000 rpm for 90 minutes. In the silylation step, 90 µL of N-methyl-N-(trimethylsilyl)-trifluoroacetamide (MSTFA) containing 1% trimethylchlorosilane (TMCS, Thermo-Pierce cat. no.TS-48915) was added to each sample. After incubation for 30 minutes at 37°C and 1000 rpm (Eppendorf Thermomixer), the derivatized samples were then transferred into 250μL glass inserts (Agilent) for GC-MS analysis.

### GC-MS metabolite analysis

GC-MS analysis was performed using a Pegasus 4D time-of-flight mass spectrometer (LECO) equipped with a Gerstel MPS2 autosampler and an Agilent 7890A oven. In the GC step, derivatization products were separated on an Rxi-5Sil^®^ column (30m, 0.25mm i.d., 0.25µm df) with an IntegraGuard^®^ pre-column using ultrapure helium at a constant flow of 0.9mL/minute as carrier gas. A split injection and injector port temperature of 240°C were employed, while the transfer line was set at 280°C. A 1µL sample volume was injected at an appropriate split ratio. The linear temperature gradient for GC started with a one-minute hold of the GC column at oven temperature of 70°C, which was then ramped at 10°C/minute to a final temperature of 300°C. Isothermal heating at 300°C was maintained for 5 minutes, followed by a final ramp to initial conditions. In the MS step, mass spectra were obtained using electron impact ionization at 70eV and a scan rate of 17 spectra/sec. Peak alignment and spectrum comparisons were carried out using the Statistical Compare feature of the ChromaTOF^®^ software (LECO).

### Metabolite identification

Identification of spectral peaks was performed by comparing their mass spectra against the built-in Fiehn primary metabolite library (Kind et al., 2009). For initial identification, a cutoff similarity score of 600 was used. Tentatively identified signals were subsequently manually validated using an in-house library of authentic standards established at the Institute of Biological Chemistry at Washington State University. Additionally, compounds with a similarity score above 700 and plausible identity were accepted as tentatively identified. The signal for the internal standard and the initial tissue weight were used for normalization.

### Statistical analysis

Data analysis was performed using the online software Metaboanalyst 5.0 (http://www.metaboanalyst.ca, Chong et al., 2019), focusing on the following questions. (1) What metabolite signatures characterize the primary (local) host response to each strain of PVY? (2) What metabolite alterations can be considered promising biomarker candidates for PVY infection? (3) Are these differential metabolite signatures time-dependent? (4) To what extent is the host metabolome response a function of the PVY strain and cultivar? (5) What metabolic pathways are associated with the differential response to PVY? To address each question, a CSV (comma-separated values) file with the relevant subset of peak intensity data was uploaded to MetaboAnalyst. Each uploaded dataset was then normalized by autoscaling before analysis with the recommended software module. To characterize the differential metabolite signatures of the primary (local) host response to each strain of PVY, the volcano plot (with fold-change threshold >1.5 and FDR [False Discovery Rate] *p*-values < 0.05) was used to perform pairwise comparisons of the host metabolome of healthy (mock-inoculated controls) and virus-inoculated leaves of each cultivar-strain combination. The variable importance in projection (VIP) score derived from the partial least-squares discriminant analysis (PLS-DA) model was then used to screen the differentially accumulated metabolites (DAMs) for candidate biomarker status, whose significance was further evaluated through one-way analysis of variance (ANOVA). The overall (global) temporal dependence of the observed metabolite signatures (profiles) was then assessed in two steps. For the first step, ANOVA-simultaneous component analysis (ASCA) (Smilde et al., 2005) was used to compare subsets of metabolomes obtained from inoculated (local) and systemically infected samples of each cultivar. To validate the ASCA results, complementary analysis with two-way within-subject ANOVA was applied to the same subset of data, followed by pattern visualization with interactive principal component analysis (iPCA). To compare the metabolic pathways related to the differential expression profiles in each potato cultivar, lists of altered compounds were uploaded to the Pathway Analysis module on Metaboanalyst, and analyzed with default parameters, the Kyoto Encyclopedia of Genes and Genomes (KEGG) pathway database and model metabolome for rice. To assess the influence of strain-cultivar interaction on the host metabolic response to PVY strain, two-way independent ANOVA was employed, at the default FDR and adjusted *p*-value of 0.05) to a further subset of the data, considering strain and cultivar as independent factors.

## Results

### GC–MS metabolite coverage

The peaks that were consistently detected and aligned by the processing software in the two cultivars comprised of metabolites belonging to various compound classes, including vitamins, esters, amino acids and polyamines, organic acids, sugars, sugar alcohols (polyols) and sugar phosphates. For statistical analysis, only metabolites that were confirmed by the standard as well as those deemed tentatively identified based on similarity score were used, including amino acids (20), organic acids (11), and the sugars and their derivatives (14).

### In potato, both common and strain-specific metabolites are induced by inoculation with potato virus Y

**Figure 1:**
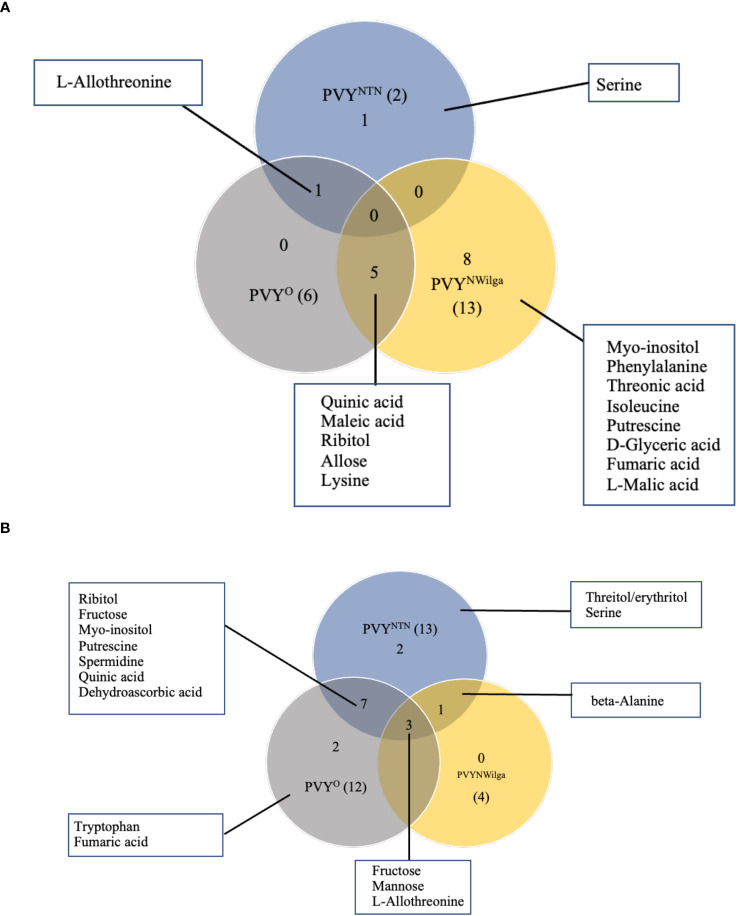
Distribution of metabolites that are changed or differentially accumulated following infection by potato virus Y. **(A)** Differentially accumulated metabolites (DAMs) detected in **(A)** Premier Russet, and **(B)** Russet Burbank. Numbers in parentheses denote the total DAM number induced by each PVY strain. A fold-change threshold >1.5 and FDR *p*-values < 0.05 was used.

Metabolites that were differentially accumulated between mock and PVY-inoculated plants are presented in **Figure 1** (and **Supplementary Figure S1**; **Supplementary Tables S1–S6**). In Premier Russet, the smallest number of DAMs (2) was detected under PVY^NTN^ inoculation, compared to six and 13 under PVY^O^ and PVY^N-Wi^ inoculation, respectively (**Figure 1A**). Consequently, limited overlap was observed between DAM sets involving PVY^NTN^ and PVY^N-Wi^ and/or PVY^O^. The major overlap was between PVY^N-Wi^ and PVY^O^, where five of the DAMs obtained under PVY^N-Wi^ inoculation matched the differential metabolite set detected after PVY^O^ inoculation. Besides this overlap between PVY^N-Wi^ and PVY^O^, a significant number of DAMs detected in Premier Russet (8) was specific to PVY^N-Wi^ infection.

In Russet Burbank, the differential response was more pronounced under PVY^NTN^ and PVY^O^ inoculations (13 and 12 DAMs, respectively), compared to the four DAMs induced by PVY^N-Wi^ inoculation (**Figure 1B**). Unlike Premier Russet where the major overlap in DAM profiles was between PVY^N-Wi^ and PVY^O^, the main DAM overlap in Russet Burbank (six metabolites) was between PVY^NTN^ and PVY^O^. In addition, alterations in three other metabolites–L-allothreonine, mannose and fructose–were induced by all three strains of PVY.

Another aspect of the differential responses was the direction of changes observed in each cultivar. In Premier Russet, for example, several amino acids were downregulated by inoculation with PVY^N-Wi^ (isoleucine and lysine) and PVY^O^ (lysine), while the majority of DAMs were upregulated. On the other hand, all the DAMs detected in Russet Burbank were enriched across the three strains of PVY (**Supplementary Figure S1**).

### Virus-induced metabolites can discriminate infections of potato virus Y in potato

**Figure 2:**
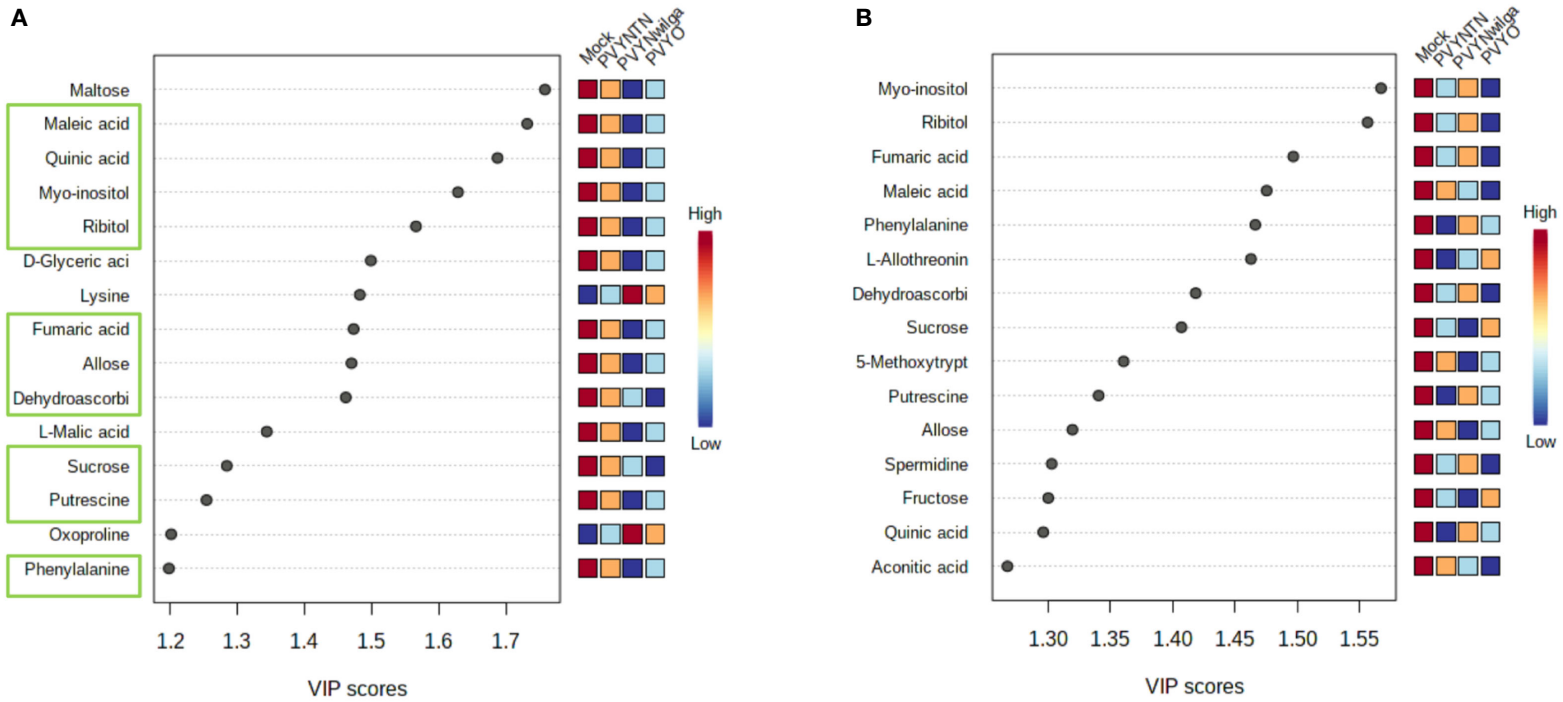
Important features selected by Partial Least Squares Discriminant Analysis (PLS-DA). **(A)** Top 15 features that discriminate infections of potato virus Y (PVY) in Premier Russet, and **(B)** Russet Burbank. Discriminatory metabolites that overlap between the two cultivars are highlighted in green boxes **(A)**.

While the initial question was that of identifying what metabolite signatures characterize the primary (local) host response to each strain of PVY, of additional interest was whether these altered metabolites can be considered promising candidate biomarkers of PVY infection. Candidate biomarker potential of the DAMs was evaluated with PLS-DA (VIP>1) followed by one-way ANOVA (*p*<0.05). The resulting VIP plots showing the top 15 metabolites detected in each cultivar are presented in **Figure 2** (and **Supplementary Tables S7, S8**).

As shown below (**Figure 2A**), the 15 most informative metabolites detected in Premier Russet were maltose, maleic acid, quinic acid, myo-inositol, ribitol, D-glyceric acid, lysine, fumaric acid, allose, dehydroascorbic acid, L-malic acid, sucrose, putrescine, oxoproline, and phenylalanine. Of these, allose, dehydroascorbic acid, fumaric acid, maleic acid, myo-inositol, phenylalanine, putrescine, quinic acid, ribitol and sucrose overlapped with the feature list obtained for Russet Burbank (**Figure 2B**). Therefore, maltose, D-glyceric acid, L-malic acid, lysine, and oxoproline were unique to Premier Russet, while L-allothreonine, 5-methoxytryptamine, spermidine, fructose and aconitic acid were specific to PVY infection of Russet Burbank.

Based on the subsequent ANOVA results, however, only quinic acid demonstrated significant difference between the mock and PVY, as well between PVY^NTN^ and PVY^N-Wi^ inoculations in Premier Russet. In the case of Russet Burbank, ANOVA selected L-allothreonine, myo-inositol and phenylalanine as the most significant features for discriminating mock versus PVY infection (**Supplementary Tables S9, S10**).

### PVY-induced pathways diverge markedly between Premier Russet and Russet Burbank

**Figure 3:**
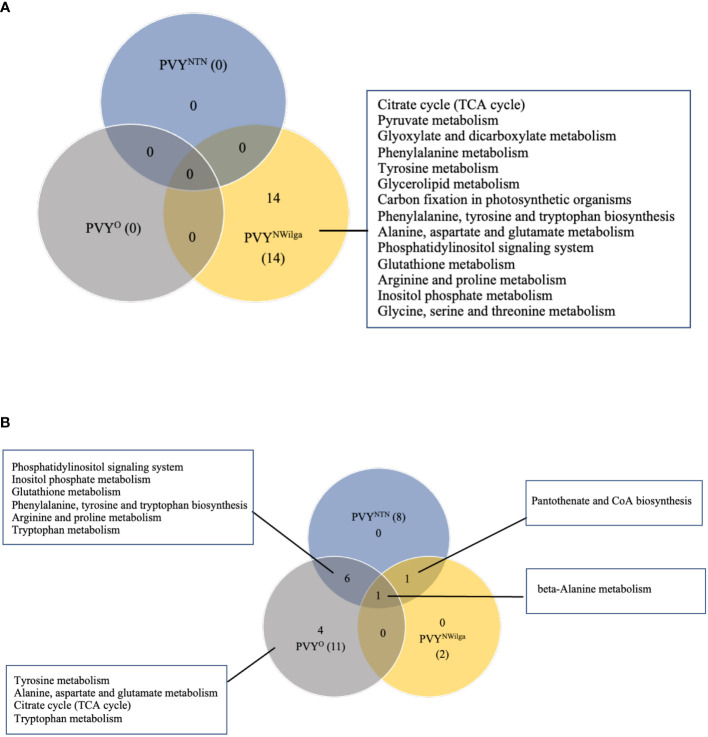
Distribution of meaningful metabolic pathways perturbed by local infection of potato with potato virus Y (PVY) (Impact>1). **(A)** pathway alterations detected in Premier Russet, and **(B)** in Russet Burbank. Pathway Analysis was performed with default parameters, KEGG pathway database and model metabolome for rice.

After showing that PVY inoculations induce common and strain-specific differential metabolite profiles in potato, KEGG (Kyoto Encyclopedia of Genes and Genomes) analysis was performed to delineate the likely underlying metabolic pathways in each cultivar. The resulting distribution of KEGG pathway perturbations is presented in **Figure 3** (and **Supplementary Tables S11–S16**; **Supplementary Figure S2**). In Premier Russet, no meaningful pathway alterations (Impact>0) were detected for PVY^NTN^ and PVY^O^, while 14 significant pathway alterations were detected in PVY^N-Wi^ infection (**Figure 3A**). In the PVY-susceptible cv. Russet Burbank, a total of 12 altered pathways were detected. Of these, only beta-alanine metabolism was found to be perturbed by all three strains of PVY (**Figure 3B**), while pantothenic acid and CoA biosynthesis was induced by both PVY^NTN^ and PVY^N-Wi^ inoculation. The main pathway overlap was between PVY^NTN^ and PVY^O^, where alterations in six metabolic pathways were observed (**Figure 3B**). Several pathway alterations were also found to be uniquely modified by PVY^O^ inoculation, including alanine, aspartate and glutamate metabolism, tryptophan metabolism, tyrosine metabolism, and tricarboxylic acid (TCA) cycle.

Based on the number of significant pathways, PVY^N-Wi^ can be considered to be metabolically more aggressive strain in Premier Russet. In Russet Burbank, PVY^NTN^ and PVY^O^ display more aggressive metabolic phenotypes than PVY^N-Wi^.

### Host responses to potato virus Y include metabolites that are subject to temporal changes

**Figure 4:**
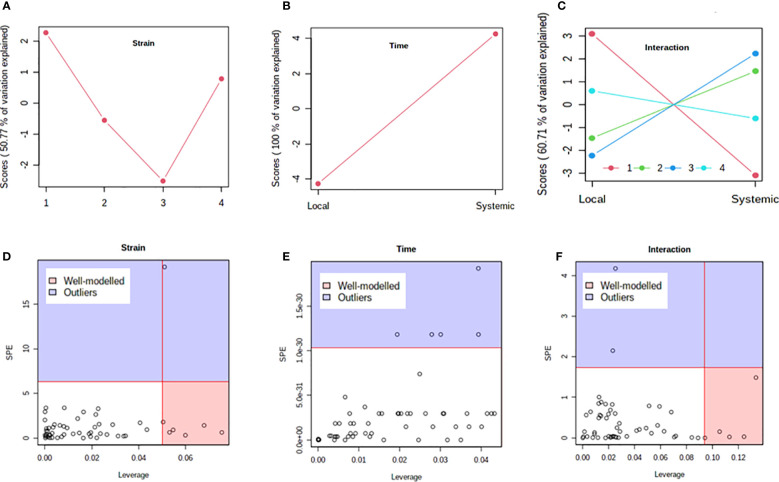
Analysis of variance – simultaneous component analysis (ASCA or ANOVA–SCA) of the differential host response to potato virus Y (PVY) infection of Premier Russet. **(A–C)** ASCA score plots showing the major patterns associated with strain, time, and their interaction; Plot legend for C: 1=mock, 2=PVY^O^, 3=PVY^N-Wi^, 4= PVY^NTN^. **(D–F)** ASCA selection of important variables (metabolites) associated with strain, time, and their interaction by leverage/squared prediction error (SPE) analysis. Vertical and horizontal lines indicate cut-off leverage and SPE values, respectively. Metabolites with a high leverage value and a low SPE value were considered to be differential metabolites (the well-modeled group). Metabolites in blue have patterns that are different from the major patterns. The list of well-modeled metabolites is given in **Supplementary Table S17**.

**Figure 5:**
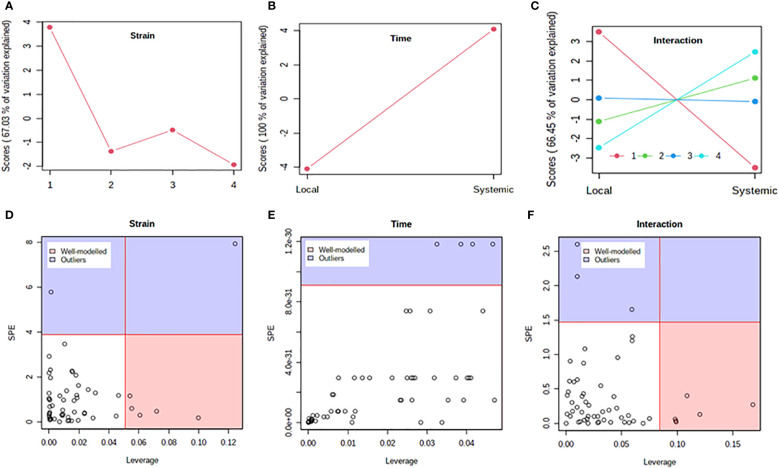
Analysis of variance – simultaneous component analysis (ASCA or ANOVA–SCA) of the differential host response to potato virus Y (PVY) infection of Russet Burbank. **(A–C)**: ASCA score plots showing the major patterns associated with strain, time, and their interaction; Plot legend for C: 1=mock, 2=PVY^O^, 3=PVY^N-Wi^, 4= PVY^NTN^. **(D–F)**: ASCA selection of important variables (metabolites) associated with strain, time, and their interaction by leverage/squared prediction error (SPE) analysis. Vertical and horizontal lines indicate cut-off leverage and SPE values, respectively. Metabolites with a high leverage value and a low SPE value were considered to be differential metabolites (the well-modeled group). Metabolites in blue have patterns that are different from the major patterns. The list of well-modeled metabolites is given in **Supplementary Table S18**.

Previous studies have shown that PVY strains can differ in translocation of virions from the foliar sites of primary infection to progeny tubers in potato. For example, PVY^N-Wi^ translocates more efficiently than PVY^NTN^ (Dupuis et al., 2019). This suggests that, while both recombinant strains ultimately result in tuber necrosis in susceptible cultivars (Karasev and Gray, 2013; Benedict et al., 2015), there may be a temporal difference in the overall host response to PVY^N-Wilga^ and PVY^NTN^ infection of potato. To test this possibility, a multi-step comparison of local and systemic response was performed using subsets of the metabolome data obtained by infection of each cultivar with the different strains of PVY. As a first step, the ASCA (or ANOVA–SCA) method was applied at a significance level of *p*<0.05. The ASCA approach blends analysis of variance (ANOVA) with Simultaneous Component Analysis (SCA, a similar method to Principal Component Analysis, PCA), thereby making the resulting interface applicable for analysis of factor relationships in multi-factor, high dimensional data (Vis et al., 2007; Bertinetto et al., 2020). The scores of the constructed ASCA sub-models are then plotted according to the first principal component (PC1) of each sub-model. Score plots showing the resulting patterns associated with strain, time (or stage) of infection, and their interaction are given in **Figure 4** (Premier Russet; **Supplementary Table S17**) and **Figure 5** (Russet Burbank; **Supplementary Table S18**)

In both cultivars, PC1 scores differed significantly between PVY-inoculated and mock-inoculated plants (**Figures 4A**, **5A**). As the time score plots show (**Figures 4B**, **5B**), the PC1 scores for local and systemic infection were also quite different. Moreover, significant strain-time interactions were detected in both cultivars (**Figures 4C**, **5C**). In this case, the initial PVY treatments (inoculations) exhibited significantly decreased score values compared to mock inoculated leaves. However, systemic infections had the opposite effect, resulting in higher PC1 scores for PVY versus mock-inoculated plants.

After ASCA analysis, leverage and squared prediction error (SPE) plots were generated to identify metabolites that follow or diverge from the trends detected by ASCA. Leverage was used to assess the importance of a metabolite to the model, while SPE was used to test the fitness of the model for a given metabolite. By this approach, metabolites with a high leverage value and a low SPE value are considered to be well-modeled and, therefore, selected as influentially affected compounds (Dai et al., 2016). In Premier Russet, six metabolites–myo-inositol, isoleucine, lysine, quinic acid, valine, and tyrosine–were well modeled by the strain factor (**Figure 4D** and **Supplementary Table S17**). Interestingly, myo-inositol, isoleucine, and quinic acid were also well modelled by the strain–time interaction, along with maltose and L-allothreonine (**Figure 5F** and **Supplementary Table S17**). In the PVY-susceptible cv. Russet Burbank, five metabolites were well modeled by the strain factor (**Figure 5D** and **Supplementary Table S18**) namely, myo-inositol, L-allothreonine, quinic acid, threonic acid, and aconitic acid. On the other hand, six metabolites had a significant contribution to the strain-time interaction, including glucose-6-phosphate (G6P), fructose-6-phosphate (G6P), tryptophan, L-allothreonine, proline, and phenylalanine (**Figure 5F** and **Supplementary Table S18**). In both cultivars, no significant features associated with time of infection were identified (**Figures 4E**, **5E**).

**Figure 6:**
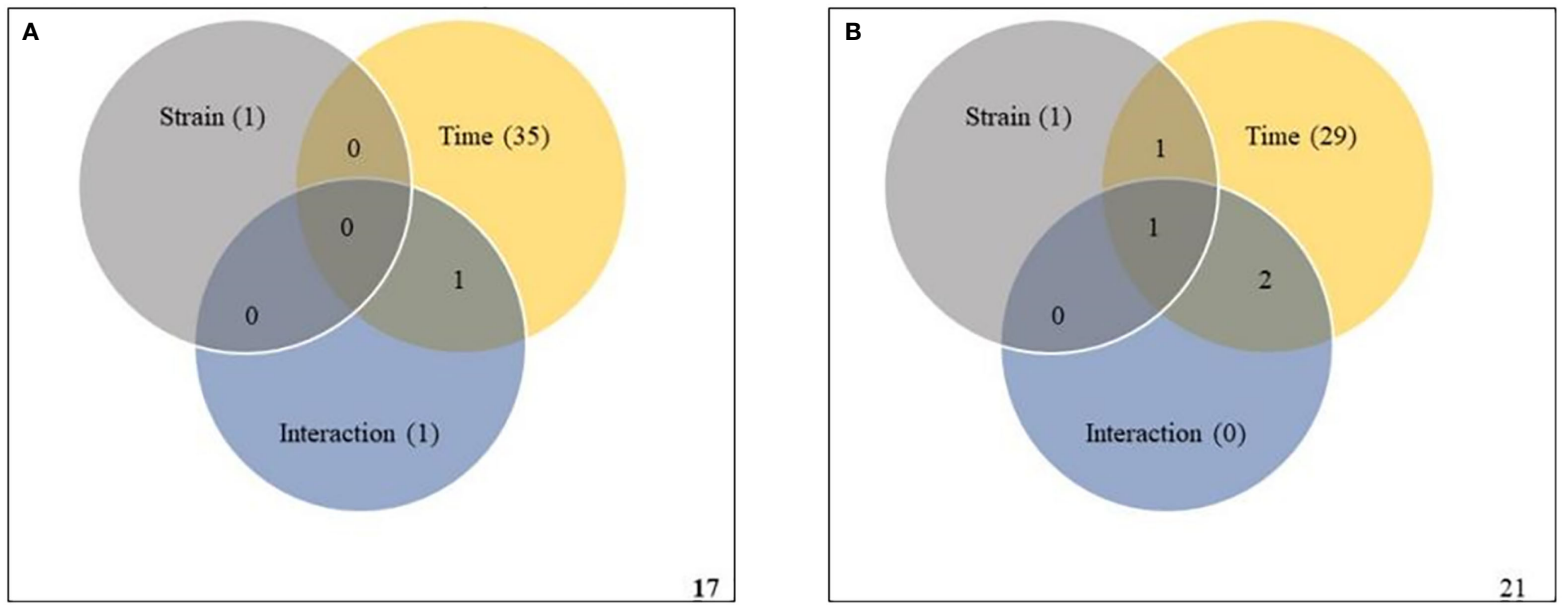
Two-way repeated measures (within subjects) analysis of variance (ANOVA) of metabolites affected by potato virus Y (PVY) strain, time and their interaction during local and systemic infection of Premier Russet **(A)** and Russet Burbank **(B)**. Grey, yellow, and blue circles respect strain, time, and their interaction, respectively. Figures in the circles represent the kinds of metabolites that responded to each element.

To validate the ASCA and SPE results, two-way within-subject (repeated) ANOVA was applied to the same subset of data to determine whether the effects of strain, infection time, and their interaction were statistically significant. A Venn diagram summary of results from the two-way ANOVA is given in **Figures 6**. In Premier Russet, the abundances of 1, 35 and 1 metabolites were significantly affected by strain, time and their interaction, respectively. Among them, the abundance of only one feature was simultaneously affected by time and strain-time interaction (**Figure 6A** and **Supplementary Table S19**). In Russet Burbank, the abundances of 1, 29 and 0 metabolic features were significantly affected by strain, time and their interaction, respectively. Of these, the abundance of only one metabolite was simultaneously affected by strain, time and their interaction (**Figure 6B** and **Supplementary Table 20**).

### Host responses to potato virus Y include metabolite changes that are strain- and cultivar-dependent

**Figure 7:**
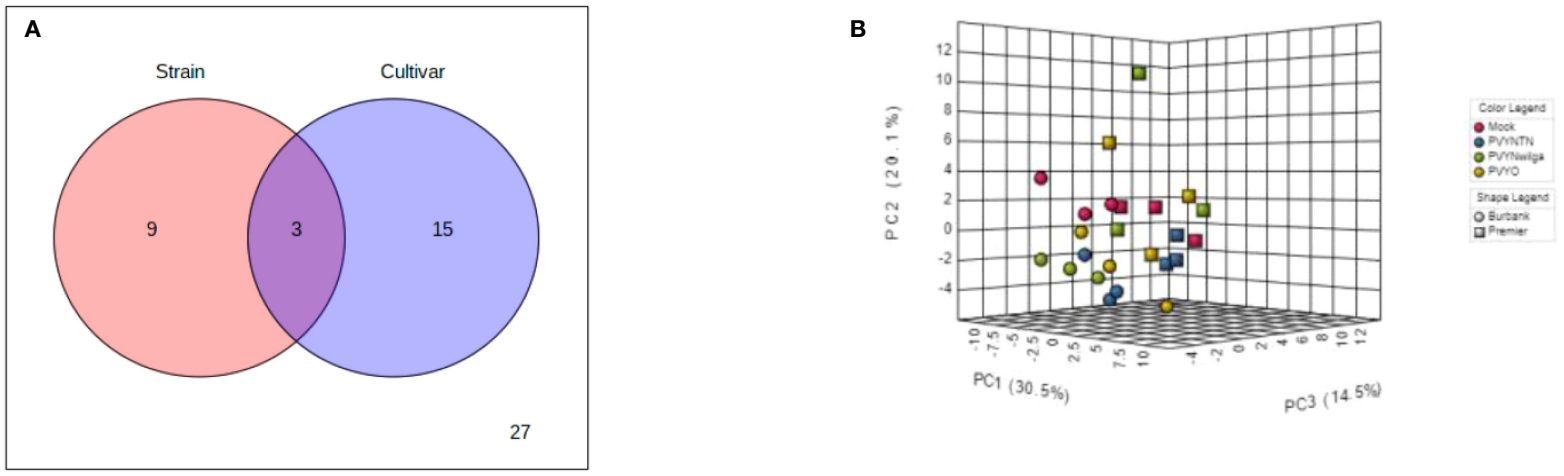
**(A)** Distribution of significant features associated with potato virus Y (PVY) strain, cultivar, and their interactions as identified by two-way analysis of variance (ANOVA). The default FDR (False Discovery Rate) and adjusted *p*-value of 0.05 were used. **(B)** Integrated Principal Components Analysis (iPCA) of PVY strain and cultivar. iPCA score plot indicates significant clustering and general grouping by strain and cultivar.

To investigate the influence of strain-cultivar interaction on the host metabolic response to PVY, two-way ANOVA was first applied to a subset of the original data obtained under local infection, considering PVY strain and cultivar as independent factors. As shown in **Figure 7A** (and **Supplementary Table S21**), the abundances of 12 and 18 metabolites were significantly affected by strain and cultivar, respectively. Among these, 3 metabolites were simultaneously affected by the interaction between strain and cultivar. An iPCA scatter visualization of this interaction is given in **Figure 7B**. As shown, the first three PCs explained nearly 70% of the total variation. Moreover, the iPCA scatterplots showed palpable separation of the mock and PVY treatments, as well as the metabolic responses in Premier Russet and Russet Burbank.

## Discussion

Regardless of virus–host compatibility, drastic modifications of host metabolism are known to occur during plant-virus interactions (Handford and Carr, 2006). As a result, deciphering how these changes influence plant resistance (or susceptibility) to the virus infection remains an important area of research. Besides contributing to the general understanding of plant-virus interaction, such research may uncover the mechanisms underlying induced disease resistance, thereby pinpointing targets that can be artificially manipulated to improve resistance to plant viruses.

In this study, the response of PVY-resistant cv. Premier Russet and susceptible cv. Russet Burbank to different PVY strains was investigated at the metabolome level. Untargeted metabolic profiles of the two cultivars were obtained after local (mechanical) inoculation and systemic infection with PVY^NTN^, PVY^N-Wi^, and PVY^O^. A mix of univariate and multivariate analysis tools were then used to explore key aspects of the host-virus interaction, including differential metabolic outcomes of PVY infection and the underlying pathway alterations, temporal characteristics, their biomarker potential, as well as aspects of strain-cultivar interaction.

In terms of morphological outcomes, the types, onset, and progression of PVY-induced foliar symptoms occurred as described previously (Goyer et al., 2015; Whitworth et al., 2021), albeit in a strain- and cultivar-dependent manner. In Premier Russet, for example, inoculations with PVY^O^ and PVY^N-Wi^ developed HR, while inoculations with PVY^NTN^ or mock control did not lead to HR (**Supplementary Figure S3**). In potato, HR resistance to PVY is conferred by *N* genes (Galvino-Costa et al., 2012.). Therefore, the observed HR phenotypes support a role for an *N*-gene that confers this resistance trait in Premier Russet, as suggested previously (Goyer et al., 2015). Another *N-*gene, *Nztbr*, was shown to confer HR against PVY^NTN^ (Kerlan et al., 2011). Therefore, the lack of HR response to PVY^NTN^ in Premier Russet precludes the presence of *Nztbr* in this cultivar, which may partly explain its reported susceptibility to PVY^NTN^ (Goyer et al., 2015).

Besides limiting a pathogen to its entry site, HR also activates the transport of defense signals that trigger systemic acquired resistance (SAR) in distant, pathogen-free parts of plants (Ádám et al., 2018). This broad-spectrum resistance mechanism leads to a *de novo* production of compounds that reduce or inhibit further attack (Heil and Bostock, 2002). Therefore, an effective SAR must limit virus replication and/or spread (Pennazio and Roggero, 1998). Hence, the lack of systemic symptoms in Premier Russet may point to the action of an effective SAR against PVY^O^ and PVY^N-Wi^. Considering the non-specificity of SAR, the effectiveness of the SAR induced by PVY^O^ and PVY^N-Wi^ in reversing the susceptibility to PVY^NTN^ seems worthy of further investigation.

Whereas HR could be considered as the hallmark of the resistance mechanism in Premier Russet, this form of resistance was not observed in Russet Burbank. By contrast, all three PVY strains induced foliar chlorosis accompanied by relatively mild lesions in this cultivar (**Supplementary Figure S4**). Unlike HR, such lesions did not restrict virus spread, thereby resulting in successful systemic infection. The resulting mosaic, rugosity, and chlorosis on younger, uninoculated leaves (**Supplementary Figure S5**) are associated with extensive disruptions to the plant photosynthetic apparatus (Pompe-Novak et al., 2001).

Differentially accumulated metabolites (DAMs) detected after PVY inoculation were annotated mainly as amino acids, organic acids, sugars, and their derivatives. Following virus infection, such shifts in primary metabolism are proposed to contribute to sustaining viral proliferation and host defense responses, thereby modulating the final outcome (susceptibility or resistance) of the infection. Overall, both common as well as strain-specific DAMs characterize the metabolic response of potato to PVY inoculation.

In Premier Russet, for example, the main overlap was between DAM sets induced by PVY^N-Wi^ and PVY^O^, which included quinic acid, maleic acid, ribitol, allose, and lysine. This may be expected, considering that PVY^N-Wi^ and PVY^O^ share other important features, including genome content and serological reactivity (Karasev et al., 2010), and a reported incompatibility with Premier Russet. Interestingly, ribitol is generally not thought to occur in plants and has previously been used as internal standard for GC-MS studies in potato (Odgerel and Ba ínfalvi, 2021). Therefore, the ‘ribitol-like’ MS spectrum and chromatographic behavior observed in this study was probably due to the presence of a similar sugar alcohol rather than ribitol itself. Indeed, sugar alcohols are known to play several significant roles in plant stress tolerance that suggest their involvement in potato-PVY interaction. For example, sugar alcohols are known for their ability to scavenge reactive oxygen species (ROS) (Miller et al., 2010), whose rapid accumulation is generally considered to be a hallmark of plant virus infection.

In comparison to PVY^O^ and PVY^N-Wi^, the differential response to PVY^NTN^ inoculation was quite limited. This is surprising, given the hitherto reported compatibility of PVY^NTN^-Premier Russet interaction. In fact, previous work (Goyer et al., 2015) has shown that compatible (susceptible) reactions involving PVY^NTN^ display far larger number of differentially expressed genes than the incompatible reactions. Therefore, the attenuated differential response to PVY^NTN^ may likely reflect a modified (or decreased) host susceptibility due to relatively advanced development stage of plants used for inoculation. This is analogous to resistance phenomena where the expression of resistance genes can be modified by, for example, development stage, plant tissue, and the environment (Vale et al., 2001). Taken more generally, the limited differential response to PVY^NTN^ may be indicative of a more complex control of its compatibility with Premier Russet.

A significant subset of metabolite alterations in Premier Russet were also found to be unique to PVY^N-Wi^ infection. In the PVY-susceptible cv. Russet Burbank, the main overlap in DAMs profiles observed was between PVY^NTN^ and PVY^O^. In both cultivars, minimal DAM overlap was found between PVY^N-Wi^ and PVY^NTN^, although both strains induce tuber necrosis in susceptible cultivars. For these reasons, it seems that PVY^N-Wi^ necrosis may be mechanistically distinguishable from that of PVY^NTN^.

In terms of the likely metabolic pathways associated with the differential profiles detected in this study, KEGG pathway analysis showed that overall, PVY-induced pathways diverge markedly between Premier Russet and Russet Burbank. A total of 15 altered pathways were annotated in Premier Russet, and 21 in Russet Burbank. Among these, four pathway alterations were specific to Premier Russet namely, glycerolipid metabolism, glyoxylate and decarbonate metabolism, carbon fixation in photosynthetic organisms, and pyruvate metabolism. Glycerolipid metabolism has been shown to play an important role in plant defense (Lim et al., 2017), including modulation of defense gene expression (Kachroo et al., 2004). Glyoxylate and dicarboxylate metabolism are known to play vital roles in energy supply under stress (Wang et al., 2010). More recent research has also demonstrated the involvement of dicarboxylate metabolic pathway proteins in the *Arabidopsis thaliana* interactome of potyviral Nia protein (Martínez et al., 2016). Carbon fixation in photosynthetic organisms, the other significantly enriched pathway, is involved in photosynthesis, pyruvate metabolism, glycolysis and the pentose phosphate pathway (Wong et al., 2014). Taken together, these findings highlight the significance of plant defense gene activation, virus-host protein-protein interactions, and energy balance in the resistance response to PVY.

Several pathways were also specifically modified in Russet Burbank, including β-alanine metabolism, pantothenate and CoA (coenzyme A) biosynthesis, and tryptophan metabolism. In plants, β-alanine acts both as a direct defense compound, as well as a precursor to pantothenate and CoA (Parthasarathy et al., 2019), which plays a central role in protein, carbohydrate and lipid metabolism. On the other hand, tryptophan and its indolic precursors are sources the plant hormone indole-3-acetic acid (IAA) as well as important defense-related metabolites such as indole glucosinolates and indolic phytoalexins (Hull and Vij, 2000). Thus, perturbations in these pathways are likely to be important in the processes underlying the PVY compatibility in Russet Burbank.

Interestingly, pathway significance did not always reflect the DAM profiles detected in Premier Russet. For example, the DAM profiles detected in response to PVY^NTN^ and PVY^O^ infection did not result in meaningful pathway alterations (**Figure 3A**). On the other hand, at least 14 significant pathway alterations were detected specifically in PVY^N-Wi^ infection. This is consistent with the differential expression profiles shown in **Figure 1**, further supporting the notion of a unique pathogenicity of this strain.

In Russet Burbank, the pathway overlap between PVY^NTN^ and PVY^O^ correlates with the differential expression pattern shown in **Figure 1**. This overlap includes phosphatidylinositol signaling system, inositol phosphate metabolism, glutathione metabolism, phenylalanine, tyrosine and tryptophan biosynthesis, arginine and proline metabolism, and phenylalanine metabolism. Functionally, phosphoinositide signaling is known to be involved in activating basal defense gene expression and priming of systemic defense responses (Hung et al., 2014). Of similar importance are inositol phosphates, which play critical roles in basal defense and signal transduction during the viral infection (Murphy et al., 1999; Loewus and Murthy, 2000). On the other hand, glutathione metabolism plays a central role in the control of ROS levels in plants (Foyer and Noctor, 2011). Meanwhile, phenylalanine, tyrosine, and tryptophan, in addition to their role as building blocks for protein synthesis, also serve as precursors of important secondary metabolites, some of which are related to plant defense (Maeda and Dudareva, 2012). Arginine and proline metabolism participate in the biosynthesis of the amino acids arginine and proline, which play important roles in stress response in plants. For example, arginine is an essential requirement for the replication of viruses and progression of viral infections. On the other hand, plants often utilize proline as a ROS quencher, thereby abrogating oxidative burst–a common response under biotic and abiotic stress (Buchanan et al., 2000; Hayat et al., 2012). The metabolism of phenylalanine plays a central role in the channeling of carbon from photosynthesis to the biosynthesis of phenylpropanoids (Pascual et al., 2016), many of which are known to function as physical and chemical barriers to infection (Dixon et al., 2002).

Interestingly, only pantothenate and CoA biosynthesis overlapped between PVY^NTN^ and PVY^N-Wi^ infection. In addition, pyruvate metabolism and citrate (or TCA) cycle, both energy related, as well as alanine, aspartate and glutamate metabolism, tryptophan metabolism, and tyrosine metabolism were unique to PVY^O^. In summary, the plant–PVY compatibility in Russet Burbank may be determined by the rebalance of metabolic pathways related to amino acid, energy, and fatty acid metabolism.

The other question considered by this study was how the host metabolome response is affected over time. For this, the study compared the metabolomes obtained from local (7dpi) and systemic infection (21dpi) of each cultivar with PVY^NTN^, PVY^N-Wi^ and PVY^O^. As modelled by ASCA, metabolite alterations in the two cultivars appeared to be significantly affected by both strain and strain–time interaction. In both cultivars, metabolite abundance decreased at 7dpi, but increased in systemic leaves. This trend reversal is consistent with previous metabolome and gene expression studies on potato leaves inoculated with PVY^N^ and PVY^NTN^ (Goyer et al., 2015; Kogovšek et al., 2016). However, leverage and SPE analysis identified only a limited number of metabolites as well-modeled by the observed trends. In Premier Russet, for example, only myo-inositol, isoleucine, quinic acid, lysine, valine, tyrosine were considered to be significantly affected by strain factor. On the other hand, L-allothreonine, quinic acid, isoleucine, maltose, and myo-inositol were affected by strain–time interaction. In the PVY-susceptible cv. Russet Burbank, myo-inositol, L-allothreonine, quinic acid, threonic acid and aconitic acid were considered to be influentially affected by strain factor. At the same time, G6P, F6P, tryptophan, L-allothreonine, proline, and phenylalanine were affected by strain–time interaction. Taken together, these temporal patterns, while limited, may act as footprints of dynamic metabolic adjustments that drive the susceptibility (or resistance) to PVY infection in potato. Moreover, the strain-dependent features of the PVY response highlight the importance of engineering broad-spectrum resistance for effective management of PVY in potato.

Another question explored by this study was the extent to which the host metabolome response is a function of the interaction between PVY strain and cultivar. In addition to DAM profiling, compatibility screening for both general and synergist effects of cultivar and strain could highlight crucial elements of PVY pathogenicity which are attributable to precise interactions between cultivar–strain specific combinations. To test this possibility, two-way ANOVA was applied specifically to data obtained under local infection, considering strain and cultivar as independent factors. As shown in **Figure 7A** (and **Supplementary Table S21**), the abundances of 12 metabolites were affected by the strain factor. This indicates that in both cultivars, these metabolites had dissimilar patterns of change over the different strains, likely reflecting a generic response of potato to PVY. On the other hand, 18 metabolites were affected by cultivar, indicating that these metabolite alterations differed in the two cultivars. Only three metabolites (tryptophan, L-allothreonine, ribitol) were affected by the interaction between strain and cultivar, which indicates that the patterns of change under the three strains differed between the two cultivars. Moreover, the iPCA scatterplots clearly separated the mock treatment, while clustering the PVY treatments during local infection of Russet Burbank. In Premier Russet, iPCA clearly separated the mock and PVY treatments along the PC2. As a whole, these observations suggest that the metabolic responses to PVY depend partly on the main effects of strain and cultivar, and partly on the precise potato cultivar–strain combination. Nonetheless, the differences in the metabolic profiles of the two cultivars show substantial concordance with the known genetic resistance–susceptibility dichotomy between them (Goyer et al., 2015).

Within the amine/amino acid pool, the major differences between the two cultivars were related to the accumulation of tryptophan, L-allothreonine, 5-methoxytryptamine, 8-spermidine, and aspartic acid. Interestingly, these metabolites appeared unaltered in Premier Russet but changed in Russet Burbank where, except for aspartic acid, all were repressed. Although diametrically opposite, both patterns of accumulation may aid virus replication. For example, a preferential increase in the consumption of aspartic acid for pyrimidine biosynthesis was found to promote the replication of certain herpes simplex viruses (Grady et al., 2013). As a corollary, it may be inferred that the increased aspartic acid accumulation may be driven by increased flux through the nucleotide biosynthesis pathway, which would support the unrestrained proliferation of PVY in Russet Burbank. In this cultivar, tryptophan, besides its role as the substrate for protein synthesis, also likely serves as a precursor of important defense-related metabolites, same as reported previously (Maeda and Dudareva, 2012). However, the likely role of L-allothreonine needs further characterization.

In virus-resistant plants, accumulation of spermidine has been shown to participate in induction of apoptosis (cell death) during HR (Walters, 2000), which may be the case in the incompatible host Premier Russet. On the other hand, spermidine may be necessary for the successful virus replication in susceptible plants (Walters, 2000). Therefore, it is likely that depletion of spermidine during the compatible virus-potato interaction in Russet Burbank represents its consumption for proliferation of PVY. The other polyamine, 5-methoxytryptamine (also known as melatonin), was shown to induce pathogenesis-related genes in *Arabidopsis* (Nawaz et al., 2016), which supports the possibility that melatonin depletion may be an important factor in the PVY susceptibility in Russet Burbank.

Another set of metabolites that differed between the two cultivars were chlorogenic acid and glycerol-3-phosphate (G3P). In Russet Burbank, both esters appeared to be depleted by PVY^O^ and PVY^N-Wi^ inoculation, but unaffected under PVY^NTN^. In plants, chlorogenate compounds are known to confer pathogen resistance (Maher et al., 1994). Consequently, the depletion of chlorogenic acid in Russet Burbank would contribute to the compatibility with PVY^O^ and PVY^N-Wi^. On the other hand, the lack of activation of chlorogenic acid by PVY^NTN^ support the notion that, perhaps, this metabolite may not be under modulation by PVY^NTN^. Alternatively, the difference with PVY^O^ and PVY^N-Wi^ may be temporal in nature.

In plants, G3P was reported to contribute to both locally expressed basal resistance and SAR **(** Mandal et al., 2011). Therefore, its depletion under PVY^O^ and PVY^N-Wi^ inoculation may partly mediate the susceptible (or compatible) reaction observed in Russet Burbank. On the other hand, the role of G3P as a potent inducer of SAR makes its unaltered status in Premier Russet ratherf surprising and may warrant a more detailed investigation of its role in PVY-potato interaction.

In the present study, several organic acids namely, threonic acid, caffeic acid, alpha-ketoglutarate (αKG), D-Glyceric acid (DGA), and methylmalonic acid (MMA) were also found to be differentially altered between the two cultivars. For example, threonic acid and caffeic acid were depleted in Russet Burbank, but unaltered in Premier Russet. Both acids have strong antioxidant activities (Gutteridge, 1995; Siquet et al., 2006; Płonka et al., 2022), making them critical components of the ROS scavenging mechanisms in plants under stress. αKG (also known as 2-oxoglutarate), also a strong ROS quencher (Mailloux et al., 2009), was elevated in Russet Burbank but unchanged in Premier Russet. Nevertheless, it seems likely that the PVY sensitivity in Russet Burbank would be preceded by a net decrease in ROS scavenging. Conversely, PVY-induced ROS accumulation likely plays a dual role in Premier Russet. First, ROS might promote programmed cell death (PCD) that accompanies HR. Second, ROS may act as signals for activating SAR (Levine et al., 1994; Dat et al., 2000). Therefore, it is also likely that the steady state levels of these antioxidants reflect a transition from an up-regulated early response after remission of infection.

With DGA, differential repression between Premier Russet and Russet Burbank was observed for PVY^O^ and PVY^N-Wi^ inoculation, but not PVY^NTN^. Even though a net decrease was observed in both cultivars, DGA was significantly more decumulated in Premier Russet than in Russet Burbank. Recent research has shown that DGA undergoes up-regulation as part of plant defense response against pathogen attack (Suharti et al., 2016), and is known to contribute to ROS scavenging under stress (Chen et al., 2018).

Also unchanged by PVY^NTN^ inoculation of the two cultivars was MMA. However, PVY^O^ and PVY^N-Wi^ inoculation had the reverse effect in Premier Russet. In Russet Burbank, MMA was increased under PVY^N-Wi^ but decreased under PVY^O^ inoculation. At present, the exact role of MMA in plant-pathogen interaction remains largely unexplored.

In conclusion, these changes in organic acid metabolism highlight the importance of regulating ROS activity during PVY infection of potato, with threonic acid, caffeic acid, αKG and DGA being essential components of this regulation.

In the present study, differential virus-induced alterations in levels of several sugars were also observed that further demonstrate the divergence in PVY compatibility between Premier Russet and Russet Burbank. For instance, xylose and F6P were repressed in Russet Burbank but unchanged in Premier Russet. Accumulation of G6P was also downregulated in Russet Burbank (under PVY^O^ and PVY^NTN^) but increased in Premier Russet (under PVY^N-Wi^ infection). In plants, G6P and F6P are intermediates in starch biosynthesis, as well as substrates for glycolysis and the pentose phosphate pathway (PPP) (Neuhaus and Emes, 2000; Rende et al., 2019). Glycolysis generates energy in the form of ATP (or adenosine triphosphate), while PPP–the alternative pathway for glucose catabolism–not only generates pentose phosphates for ribonucleotides synthesis, but also NADPH. In plants, NADPH (or reduced nicotinamide adenine dinucleotide phosphate) is a substrate for fatty acid biosynthesis and production of reduced glutathione (GSH), which is a key scavenger of ROS (Nathan and Ding, 2010).

Therefore, the observed downregulation of G6P and F6P in Russet Burbank may be an indication of disruption in energy production, as well as impaired starch biosynthesis, which is considered an indicator of successful virus infection (Zhao et al., 2022). In addition, G6P and F6P depletion may indicate a repression of the glycolytic-PPP shunt. In this flux mode, decreased NADPH levels could diminish the plant’s ability to regulate cellular ROS, thus potentiating the susceptibility in Russet Burbank.

Xylose, the other sugar repressed in Russet Burbank, is a major component of hemicellulose in plant cell walls (Harper and Bar-Peled, 2002; Liu et al., 2021). Current evidence indicates that differences in xylose content of the cell wall may influence the compatibility between pathogens and their hosts (Sanchez-Rodriguez et al., 2009). Therefore, cell wall hemicellulose content and, consequently, cell wall structure may play a significant role in potato susceptibility to PVY.

As mentioned above, xylose and F6P were found to be unaltered in Premier Russet, while G6P was increased only under PVY^N-Wi^ infection. Although other interpretations are possible, the ‘steady state’ behavior of xylose and F6P may provide evidence of recovery from virus inoculation and an effective resistance mechanism in Premier Russet. A possible functional explanation for the upregulation of G6P in Premier Russet would be its participation in activation of pathogenesis-related (PR) gene expression against PVY^N-Wi^, in the same way as reported previously (Xiao et al., 2000).

Finally, we investigated whether the virus-induced metabolites may be potential candidate biomarkers of PVY infection in potato. Ideal biomarkers are inexpensive, reliable, consistent, easily measured, and their expression is altered under disease conditions (Verma et al., 2011). Using the variable importance in projection (VIP) cutoff score (VIP>1), a feature list comprised of allose, dehydroascorbic acid, fumaric acid, maleic acid, myo-inositol, phenylalanine, putrescine, quinic acid, ribitol and sucrose was identified in Premier Russet and Russet Burbank. As these metabolites were co-induced in both cultivars, they could be considered generic indicators of PVY infection in potato. On the other hand, maltose, D-glyceric acid, L-malic acid, lysine, and oxoproline were unique to Premier Russet, while L-allothreonine, 5-methoxytryptamine, spermidine, fructose and aconitic acid were specific to Russet Burbank. Given their cultivar specificity, these metabolites may be likely biomarker candidates for PVY resistance and susceptibility in potato. However, their utility requires further exploration over diverse germplasm, time-course and systemic infection, as well as tissue specificity, considering that validation with ANOVA identified quinic acid, L-allothreonine, myo-inositol, and phenylalanine as consistent discriminators of PVY infection.

## Conclusions

PVY is an economically important viral pathogen of potato. In this study, we used GC-MS to investigate how PVY-resistant and susceptible potato cultivars respond to infection by three different strains of PVY (PVY^NTN^, PVY^N-Wi^, and PVY^O^). Our analysis showed that, in general, PVY infections in potato lead to detectable changes in metabolites and pathways related to amino acid, energy, and fatty acid metabolism. In Premier Russet, a major overlap in differential metabolite sets occurred between PVY^N-Wi^ and PVY^O^, while in Russet Burbank, the main overlap was observed between PVY^NTN^ and PVY^O^. Besides some shared metabolic outcomes, meaningful pathway alterations in Premier Russet occurred only in response to PVY^N-Wi^ inoculation. Overall, limited overlap was observed between PVY^NTN^ and PVY^N-Wi^, suggesting that PVY^N-Wi^-induced necrosis may be mechanistically distinguishable from that of PVY^NTN^. Unraveling the detailed workings of these altered pathways, as well as the role of secondary metabolism could lead to the validation of a specific pathways that plant breeders could re-engineer to protect potato and other economically important PVY susceptible hosts such as tobacco, tomato and pepper against these necrotic strains of PVY. In this study, using PLS-DA and ANOVA, we identified 10 common and seven cultivar-specific metabolites as potential indicators of PVY infection and susceptibility or resistance in potato. However, their utility requires further validation across different genetic backgrounds, time-course and systemic infection, as well as for tissue specificity. Another key finding from this study was the importance of strain–time interaction in response to PVY. In this regard, G6P and F6P were significantly affected in Russet Burbank. This highlights the relevance of the regulation of carbohydrate metabolism for defense against PVY. Finally, this study also showed host responses to PVY include metabolite changes that are strain- and cultivar-dependent. In particular, the amino acid, organic acid, and sugar profiles of the two cultivars showed notable concordance with the known genetic resistance–susceptibility dichotomy between them. As a result, effective management of these different strains of PVY will likely require engineering broad-spectrum resistance in potato.

## Data availability statement

The raw data supporting the conclusions of this article will be made available by the authors, without undue reservation.

## Author contributions

HRP conceived the project and secured funding. RM, MK, LM, AB, and DRG designed the experiments. RM and AB carried out the experiments and data analysis, RM drafted the manuscript. All authors contributed to the article and approved the submitted version.
