# Supplementary material for: Pathogen-triggered metabolic adjustments to potato virus Y infection in potato
Source: Front Plant Sci. 2023 Feb 20;13:1031629. doi: 10.3389/fpls.2022.1031629 (PMC9986423; doi:10.3389/fpls.2022.1031629)
Supplement: Supplementary file 1 [file DataSheet_1.pdf]

Supplementary Material

Pathogen-triggered metabolic adjustments to potato virus Y infection in potato

1 Supplementary Data

All relevant data are within the manuscript.

2 Supplementary Figures and Tables

2.1 Supplementary Figures

frontiers

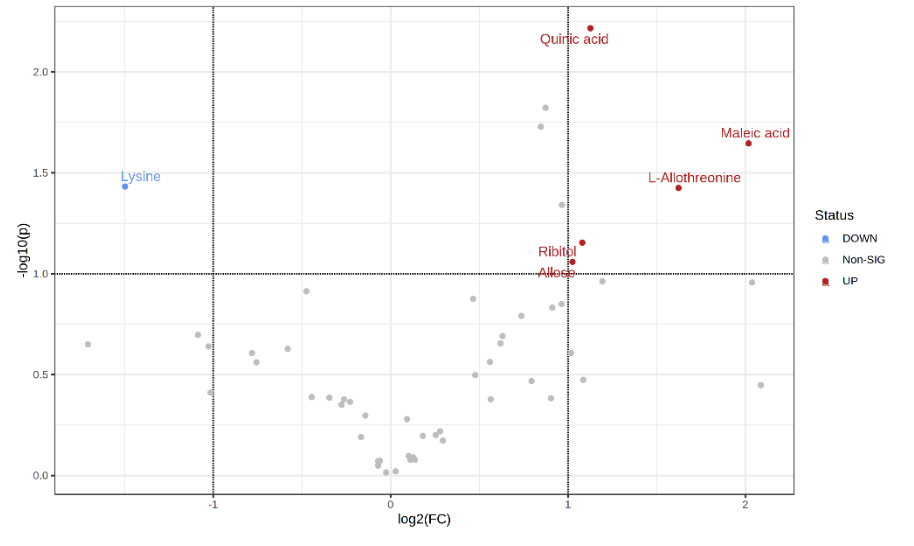

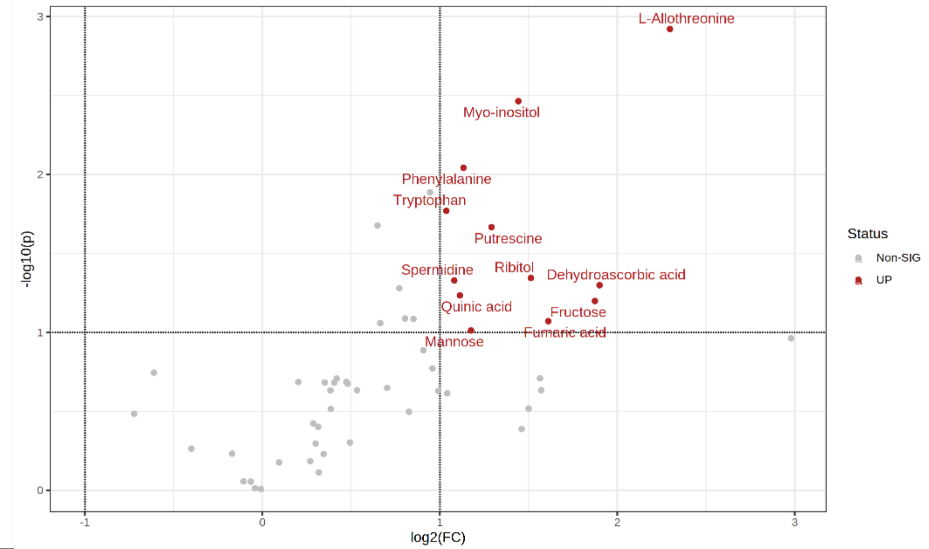

A

D

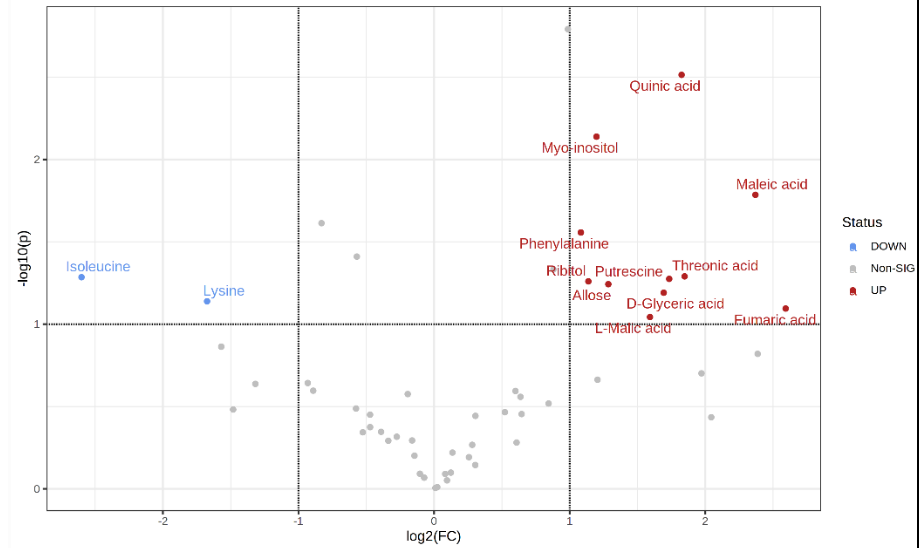

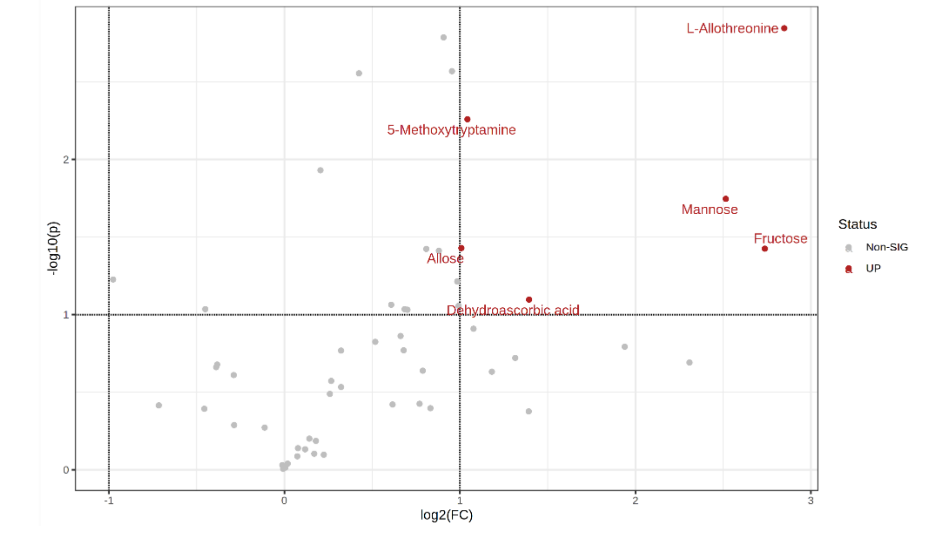

B

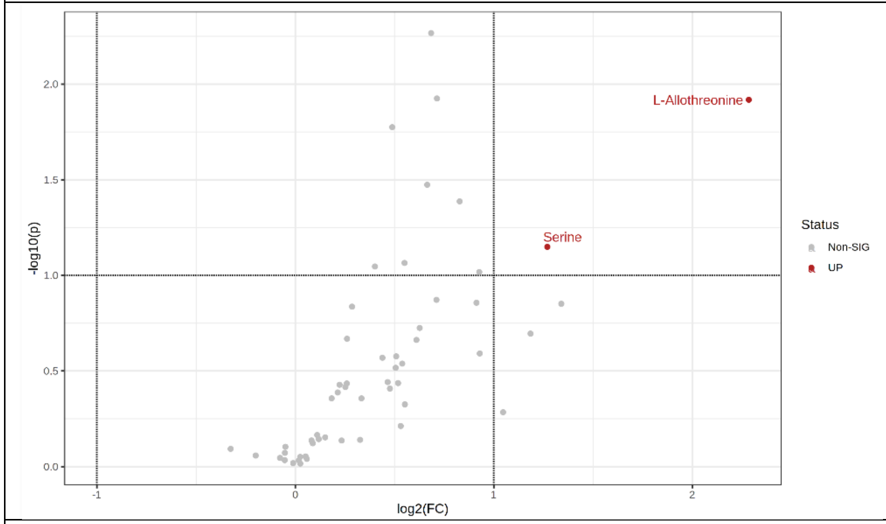

E

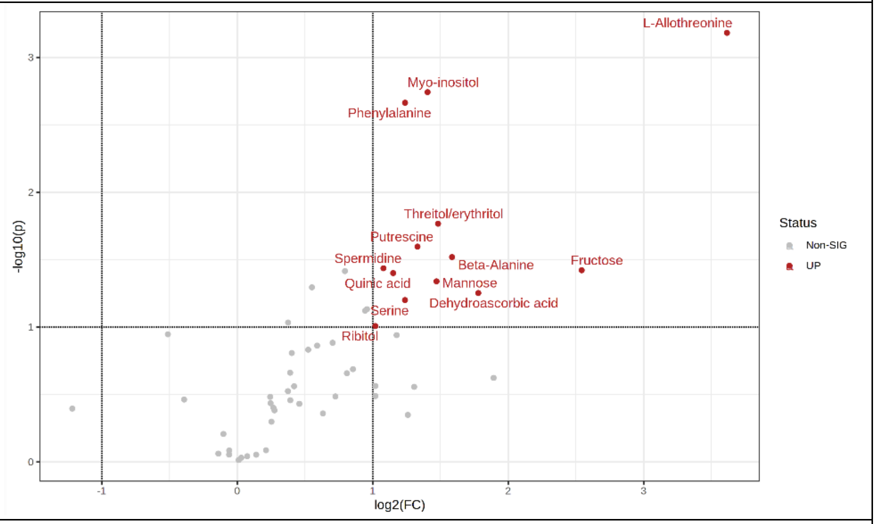

C

F

Supplementary Figure S1 Volcano plots showing the differential metabolite response in (**A-C**) Premier Russet (**A**: PVY^{NTN}, **B**: PVY^{O}, **C**: PVY$^{N\text{-}Wi}$) and (**D-F**) Russet Burbank (**D**: PVY^{NTN}, **E**: PVY^{O}, **F**: PVY$^{N\text{-}Wi}$). DAMs in red are upregulated whereas those in blue are downregulated. A fold-change threshold >1.5 and FDR *p*-values < 0.05 was used.

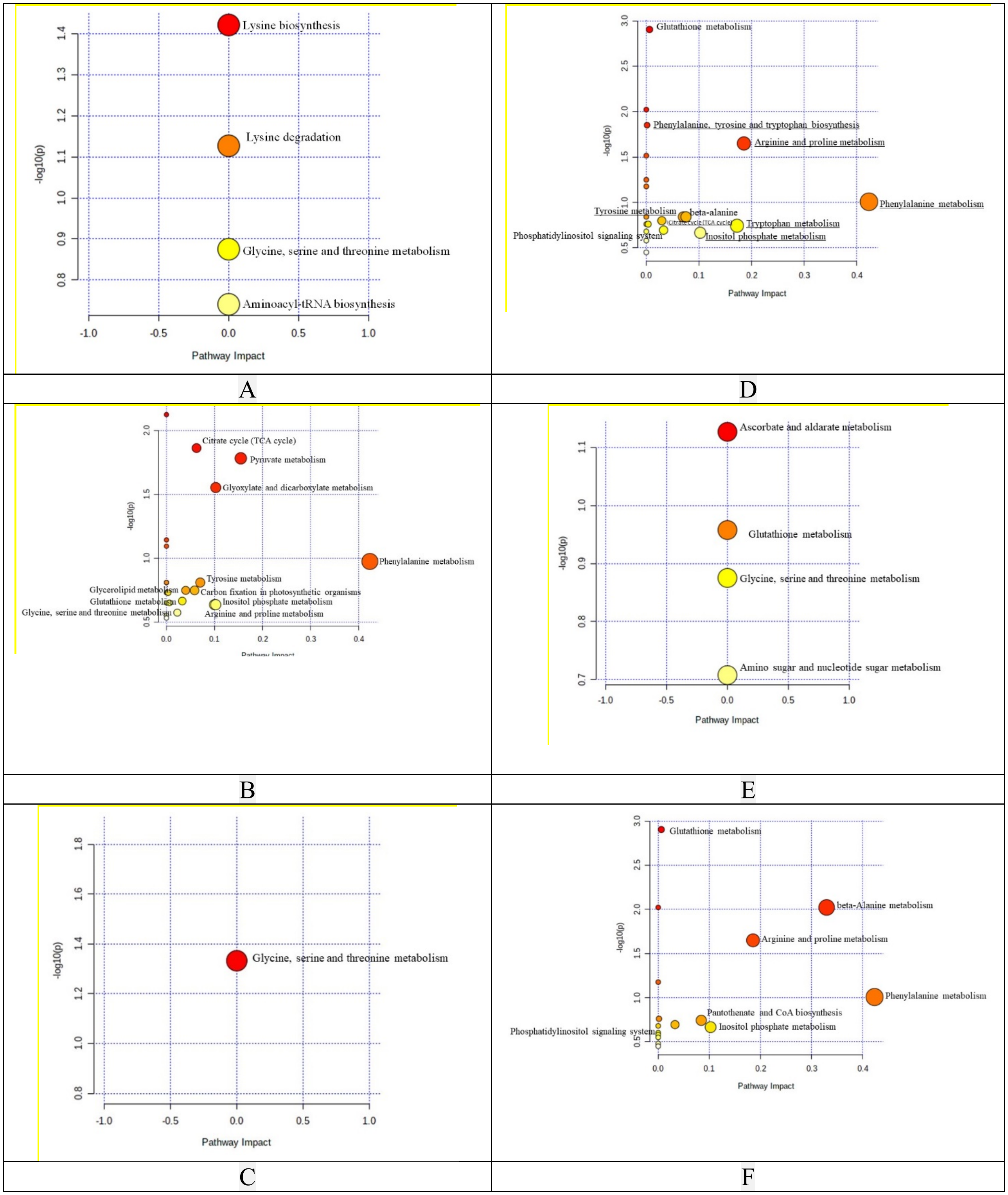

Supplementary Figure S2. KEGG pathway analysis of the differential response to PVY in Premier Russet (**A-C**) and Russet Burbank (**D-F**). Pathways disturbed by PVY^{O} (**A and D**), PVY^{NTN} (**B and E**), and PVY$^{N\text{-}Wi}$ (**C and F**). The circles represent different KEGG pathway. Impact value is calculated from pathway topology analysis by MetaboAnalyst. A bigger value indicates a pathway as more important. The size of circle represents the number of DAMs in this pathway,

and the color represents the *p*-value, which is the significance level in enrichment analysis statistics. Details of DAMs in each pathway are listed in Supplementary Tables S1-S6.

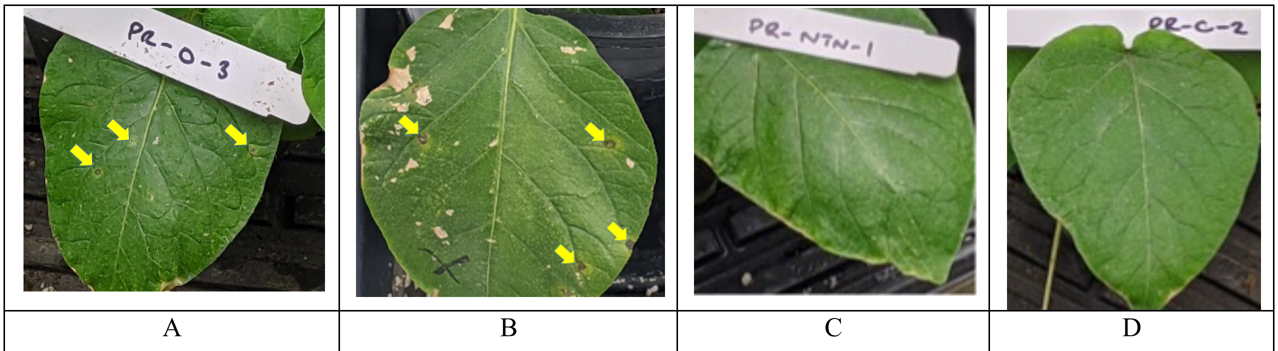

A B C D

Supplementary Figure S3. Poato virus Y-induced symptom development in Premier Russet. **A**: Hypersensitive response (HR) or spot necroses on leaf inoculated with PVY^{O} at 14 days post-inoculation (dpi); **B**: HR on leaf inoculated with $PVY^{N\text{-}Wi}$ at 21 dpi. Yellow arrows show local necrosis typical of HR. Leaves inoculated with PVY^{NTN} (**C**) and buffer (mock, **D**) displayed no symptoms.

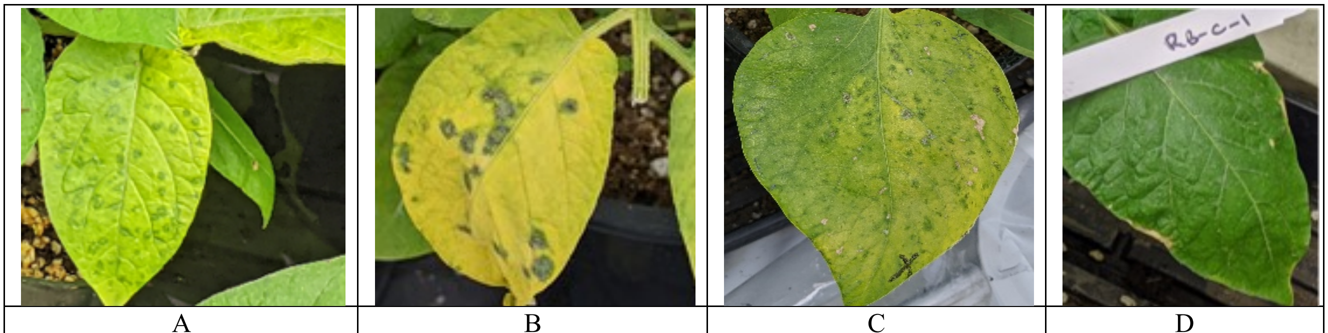

A B C D

Supplementary Figure S4. Representative images of potato virus Y (PVY)-induced symptoms on Russet Burbank. Localized necrosis and chlorosis on Russet Burbank at 21 days post-inoculation (dpi) with PVY^{O} (**A**), PVY^{NTN} (**B**), and $PVY^{N\text{-}Wi}$ (**C**). Buffer or mock inoculation (**D**) produced no symptoms.

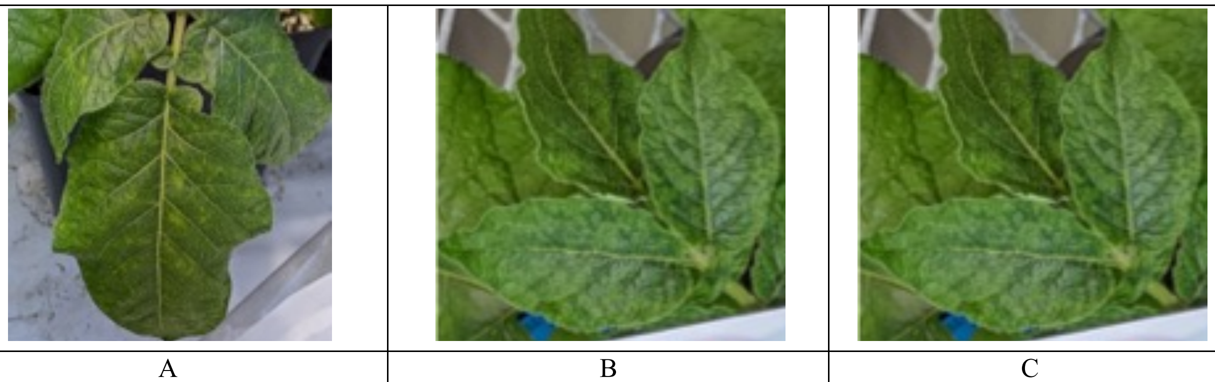

A B C

Supplementary Figure S5. Systemic symptoms induced by potato virus Y (PVY) recorded on Russet Burbank at ~42 post-inoculation (dpi). **A**: PVY^{O}-induced mosaic and chlorosis. **B**: $PVY^{N\text{-}Wi}$-induced mosaic and rugosity. **C**: PVY^{NTN}-induced mosaic and chlorosis.

2.2 Supplementary Tables

Table S1. Volcano feature details of DAMs in during PVY$^{N\text{-}Wi}$ inoculation of Russet Burbank

	FC	Variation	log2(FC)	raw.pval	-log10(p)
L-Allothreonine	7.2008	↑	2.8482	0.001427	2.8457
5-Methoxytryptamine	2.0603	↑	1.0429	0.005507	2.2591
Mannose	5.716	↑	2.515	0.017941	1.7461
Allose	2.012	↑	1.0086	0.037244	1.4289
Fructose	6.6685	↑	2.7374	0.037595	1.4249
Dehydroascorbic acid	2.628	↑	1.394	0.080073	1.0965

Table S2. Volcano feature details of DAMs in during PVY^{O} inoculation of Russet Burbank

	FC	Variation	log2(FC)	raw.pval	-log10(p)
L-Allothreonine	4.9131	↑	2.2966	0.001199	2.9212
Myo-inositol	2.7169	↑	1.442	0.003431	2.4645
Phenylalanine	2.1935	↑	1.1332	0.009071	2.0423
Tryptophan	2.0509	↑	1.0363	0.016981	1.77
Putrescine	2.447	↑	1.291	0.021537	1.6668
Ribitol	2.8539	↑	1.5129	0.045205	1.3448
Spermidine	2.1155	↑	1.081	0.046821	1.3296
Dehydroascorbic acid	3.7319	↑	1.8999	0.050231	1.299
Quinic acid	2.1629	↑	1.113	0.058296	1.2344
Fructose	3.6652	↑	1.8739	0.063248	1.1989
Fumaric acid	3.0547	↑	1.611	0.084871	1.0712
Mannose	2.2587	↑	1.1755	0.097216	1.0123

Table S3. Volcano feature details of DAMs in during PVY^{NTN} inoculation of Russet Burbank

	FC	Variation	log2(FC)	raw.pval	-log10(p)
L-Allothreonine	12.256	↑	3.6154	0.000658	3.1816
Myo-inositol	2.6484	↑	1.4051	0.00181	2.7422
Phenylalanine	2.3601	↑	1.2388	0.002171	2.6634
Threitol/erythritol	2.7945	↑	1.4826	0.017103	1.7669
Putrescine	2.5152	↑	1.3307	0.025281	1.5972
Beta-Alanine	3.0013	↑	1.5856	0.03022	1.5197
Spermidine	2.1124	↑	1.0789	0.036591	1.4366
Fructose	5.8267	↑	2.5427	0.037832	1.4221
Quinic acid	2.2209	↑	1.1512	0.039728	1.4009
Mannose	2.7721	↑	1.471	0.045753	1.3396
Dehydroascorbic acid	3.4332	↑	1.7796	0.055831	1.2531
Serine	2.3594	↑	1.2384	0.062954	1.201
Ribitol	2.0272	↑	1.0195	0.098084	1.0084

Table S4. Volcano feature details of DAMs in during PVY$^{N\text{-}Wi}$ inoculation of Premier Russet

	FC	Variation	log2(FC)	raw.pval	-log10(p)
Quinic acid	3.5441	↑	1.8254	0.003066	2.5134
Myo-inositol	2.2939	↑	1.1978	0.007273	2.1383
Maleic acid	5.1687	↑	2.3698	0.01641	1.7849
Phenylalanine	2.1174	↑	1.0823	0.027757	1.5566
Threonic acid	3.5991	↑	1.8476	0.051277	1.2901
Isoleucine	0.16488	↓	-2.6005	0.05189	1.2849
Putrescine	3.3262	↑	1.7339	0.053126	1.2747
Ribitol	2.2006	↑	1.1379	0.055004	1.2596
Allose	2.4365	↑	1.2848	0.05722	1.2425
D-Glyceric acid	3.2344	↑	1.6935	0.064443	1.1908
Lysine	0.31331	↓	-1.6743	0.072658	1.1387
Fumaric acid	6.033	↑	2.5929	0.080402	1.0947
L-Malic acid	3.0148	↑	1.592	0.090564	1.043

Table S5. Volcano feature details of DAMs in during PVY^{O} inoculation of Premier Russet

	FC	Variation	log2(FC)	raw.pval	-log10(p)
Quinic acid	2.1839	↑	1.1269	0.006074	2.2165
Maleic acid	4.0523	↑	2.0187	0.022605	1.6458
Lysine	0.35429	↓	-1.497	0.036964	1.4322
L-Allothreonine	3.0807	↑	1.6232	0.037597	1.4248
Ribitol	2.1151	↑	1.0808	0.070116	1.1542
Allose	2.0358	↑	1.0256	0.087305	1.059

Table S6. Volcano feature details of DAMs in during PVY^{NTN} inoculation of Premier Russet

	FC	Variation	log2(FC)	raw.pval	-log10(p)
L-Allothreonine	4.8731	↑	2.2848	0.012075	1.9181
Serine	2.4112	↑	1.2697	0.070942	1.1491

Table S7 Variable importance in projection (VIP) score plot for the top 15 most important metabolite features identified by PLS-DA in Premier Russet

Metabolite	Comp. 1	Comp. 2	Comp. 3
Myo-inositol	1.5671	1.3573	1.2475
Ribitol	1.5563	1.2959	1.192
Fumaric acid	1.4967	1.2488	1.1485
Maleic acid	1.4754	1.2516	1.1508
Phenylalanine	1.4663	1.2373	1.1374
L-Allothreonine	1.4628	1.2526	1.1892
Dehydroascorbic acid	1.4185	1.181	1.1178
Sucrose	1.407	1.1773	1.1151
5-Methoxytryptamine	1.3607	1.1451	1.0626

Putrescine	1.3406	1.1153	1.0611
Allose	1.3196	1.1197	1.0582
Spermidine	1.303	1.0845	0.99707
Fructose	1.3002	1.1051	1.1248
Quinic acid	1.2962	1.1114	1.0215
Aconitic acid	1.2675	1.0574	1.0161

Table S8 Variable importance in projection (VIP) score plot for the top 15 most important metabolite features identified by PLS-DA in Russet Burbank

Metabolite	Comp. 1	Comp. 2	Comp. 3
Maltose	1.7582	1.5892	1.5649
Maleic acid	1.7317	1.5727	1.5431
Quinic acid	1.6876	1.518	1.4902
Myo-inositol	1.6287	1.4626	1.4361
Ribitol	1.5662	1.4081	1.3878
D-Glyceric acid	1.499	1.3637	1.3461
Lysine	1.4826	1.3407	1.3159
Fumaric acid	1.4733	1.3811	1.3561
Allose	1.4701	1.321	1.3001
Dehydroascorbic acid	1.4616	1.3869	1.3629
L-Malic acid	1.344	1.2621	1.2464
Sucrose	1.2846	1.2436	1.2545
Putrescine	1.2543	1.1526	1.1342
Oxoproline	1.2018	1.0804	1.1055
Phenylalanine	1.1984	1.1175	1.0994

Table S9. One-way ANOVA result of significant metabolites affected by PVY inoculation in Premier Russet.

	f.value	p.value	#NAME?	FDR	Fisher's LSD
Quinic acid	16.606	0.000851	3.0703	0.045927	Mock - PVY^{NTN}; Mock - PVY$^{N\text{-}Wi}$; Mock - PVY^{O}; PVY^{NTN} - PVY$^{N\text{-}Wi}$

Table S10. One-way ANOVA result of significant metabolites affected by PVY inoculation of Russet Burbank.

	f.value	p.value	#NAME?	FDR	Fisher's LSD
L-Allothreonine	58.866	8.53E-06	5.0691	0.000461	Mock - PVY^{NTN}; Mock - PVY$^{N\text{-}Wi}$; Mock - PVY^{O}
Myo-inositol	29.098	0.000118	3.9279	0.003188	Mock - PVY^{NTN}; Mock - PVY$^{N\text{-}Wi}$; Mock - PVY^{O}
Phenylalanine	17.852	0.000665	3.1775	0.011962	Mock - PVY^{NTN}; Mock - PVY$^{N\text{-}Wi}$; Mock - PVY^{O}

Table S11 Metabolic pathways affected by PVY^{O} inoculation of Premier Russet

Pathway Name	Match Status	p	FDR	Impact	Details
Lysine biosynthesis	1/9	0.037944	1.0	0.0	KEGG
Lysine degradation	1/18	0.07468	1.0	0.0	KEGG
Glycine, serine and threonine metabolism	1/33	0.13331	1.0	0.0	KEGG
Aminoacyl-tRNA biosynthesis	1/46	0.18158	1.0	0.0	KEGG

Table S12 Meaningful metabolic pathways affected by $PVY^{N\text{-}Wi}$ inoculation of Premier Russet (Impact >0)

Pathway Name	Match Status	p	FDR	Impact	Details
Citrate cycle (TCA cycle)	2/20	0.013712	0.5224	0.06258	KEGG
Pyruvate metabolism	2/22	0.016497	0.5224	0.15462	KEGG
Glyoxylate and dicarboxylate metabolism	2/29	0.027952	0.66387	0.10252	KEGG
Phenylalanine metabolism	1/12	0.10609	1.0	0.42308	KEGG
Tyrosine metabolism	1/18	0.15515	1.0	0.07027	KEGG
Glycerolipid metabolism	1/21	0.17873	1.0	0.04	KEGG
Carbon fixation in photosynthetic organisms	1/21	0.17873	1.0	0.05846	KEGG
Phenylalanine, tyrosine and tryptophan biosynthesis	1/22	0.18646	1.0	0.0015	KEGG
Alanine, aspartate and glutamate metabolism	1/22	0.18646	1.0	0.0036	KEGG
Phosphatidylinositol signaling system	1/26	0.21669	1.0	0.03285	KEGG
Glutathione metabolism	1/27	0.22409	1.0	0.00606	KEGG
Arginine and proline metabolism	1/28	0.23142	1.0	0.09974	KEGG
Inositol phosphate metabolism	1/28	0.23142	1.0	0.10251	KEGG
Glycine, serine and threonine metabolism	1/33	0.26712	1.0	0.02252	KEGG

Table S13 Meaningful metabolic pathways affected by PVY^{NTN} inoculation of Premier Russet (Impact >0)

Pathway Name	Match Status	p	FDR	Impact	Details
Glycine, serine and threonine metabolism	1/33	0.046505	1.0	0.0	KEGG

Table S14 Meaningful metabolic pathways affected by PVY^{O} inoculation of Russet Burbank (Impact >0)

Pathway Name	Match Status	p	FDR	Impact	Details
Glutathione metabolism	3/27	0.0012472	0.11848	0.00606	KEGG
Phenylalanine, tyrosine and tryptophan biosynthesis	2/22	0.014092	0.44625	0.0015	KEGG
Arginine and proline metabolism	2/28	0.02241	0.53225	0.18548	KEGG
Phenylalanine metabolism	1/12	0.098312	1.0	0.42308	KEGG
Tyrosine metabolism	1/18	0.14407	1.0	0.07027	KEGG
beta-Alanine metabolism	1/18	0.14407	1.0	0.0754	KEGG
Citrate cycle (TCA cycle)	1/20	0.15884	1.0	0.0295	KEGG
Alanine, aspartate and glutamate metabolism	1/22	0.17338	1.0	0.0036	KEGG
Tryptophan metabolism	1/23	0.18056	1.0	0.17241	KEGG
Phosphatidylinositol signaling system	1/26	0.20177	1.0	0.03285	KEGG
Inositol phosphate metabolism	1/28	0.21563	1.0	0.10251	KEGG

Table S15 Meaningful metabolic pathways affected by PVY$^{N\text{-}Wi}$ inoculation of Russet Burbank (Impact >0)

Pathway Name	Match Status	p	FDR	Impact	Details
beta-Alanine metabolism	1/18	0.050392	1.0	0.25397	KEGG
Pantothenate and CoA biosynthesis	1/23	0.064046	1.0	0.08423	KEGG

Table S16 Meaningful metabolic pathways affected by PVY^{NTN} inoculation of Russet Burbank (Impact >0)

Pathway Name	Match Status	p	FDR	Impact	Details
Glutathione metabolism	3/27	0.0012472	0.11848	0.00606	KEGG
beta-Alanine metabolism	2/18	0.0095133	0.30126	0.32937	KEGG
Arginine and proline metabolism	2/28	0.02241	0.53225	0.18548	KEGG
Phenylalanine metabolism	1/12	0.098312	1.0	0.42308	KEGG
Phenylalanine, tyrosine and tryptophan biosynthesis	1/22	0.17338	1.0	0.0015	KEGG
Pantothenate and CoA biosynthesis	1/23	0.18056	1.0	0.08423	KEGG
Phosphatidylinositol signaling system	1/26	0.20177	1.0	0.03285	KEGG
Inositol phosphate metabolism	1/28	0.21563	1.0	0.10251	KEGG

Table S17 Metabolites in Premier Russet which were well-modeled by SPE for strain and strain-time interaction. Leverage threshold: 0.9; Alpha threshold: 0.05

Strain			Strain-time interaction		
Metabolite	Leverage	SPE	Metabolite	Leverage	SPE
Myo-inositol	0.075518	0.64108	L-Allothreonine	0.13369	1.4865
Isoleucine	0.067961	1.4282	Quinic acid	0.12451	0.029253
Quinic acid	0.059974	0.32337	Isoleucine	0.11323	0.02469
Lysine	0.054753	0.92126	Maltose	0.10566	0.15403
Valine	0.053219	0.65098	Myo-inositol	0.094306	0.000446
Tyrosine	0.050473	1.8068			

Table S18 Metabolites in Russet Burbank which were well-modeled by SPE for strain and strain-time interaction.

Strain			Strain-time interaction		
Metabolite	Leverage	SPE	Metabolite	Leverage	SPE
Myo-inositol	0.099884	0.18243	Glucose-6-phosphate	0.1683	0.27119
L-Allothreonine	0.071925	0.47443	Fructose-6-phosphate	0.12047	0.12971
Quinic acid	0.060798	0.3036	Tryptophan	0.10895	0.40092
Threonic acid	0.05528	0.60213	L-Allothreonine	0.09906	0.020825
Aconitic acid	0.054082	1.1597	Proline	0.098833	0.037564
			Phenylalanine	0.098236	0.061717

Table S19. Two-way repeated measures (within subjects) ANOVA of metabolites affected by PVY strain, time and their interaction during local and systemic infection of Premier Russet

	Strain(F.val)	Strain(raw.p)	Strain(adj.p)	Time(F.val)	Time(raw.p)	Time(adj.p)	Interaction(F.val)	Interaction(raw.p)	Interaction(adj.p)
Myo-inositol	6.9776	0.003246	0.089268	11.509	0.003719	0.007576	9.3793	0.000819	0.033847
Oxoproline	5.9526	0.006318	0.11583	100.54	2.65E-08	6.72E-07	3.6092	0.036563	0.25137
Lysine	5.0198	0.012178	0.16744	25.86	0.00011	0.000357	4.0497	0.025546	0.24702
D-Glyceric acid	3.9569	0.027518	0.25225	123.41	6.24E-09	3.43E-07	3.6858	0.034315	0.25137
Quinic acid	3.709	0.033666	0.26452	0.23064	0.63755	0.67433	8.6267	0.001231	0.033847
Tyrosine	2.8496	0.070312	0.48339	8.4751	0.010201	0.018098	0.73704	0.54517	0.74961
L-Malic acid	2.6855	0.081492	0.49243	5.6978	0.029676	0.045339	3.0539	0.058704	0.29216
Beta-Alanine	2.2112	0.12641	0.53481	19.179	0.000467	0.001426	1.47	0.26027	0.53019
Tryptophan	2.0251	0.15094	0.58834	6.0491	0.02568	0.040354	2.2323	0.12392	0.37863
Serine	1.9429	0.16338	0.58834	12.629	0.002646	0.005718	2.3121	0.11495	0.3719
Mannose	1.6168	0.22487	0.59013	28.922	6.16E-05	0.000212	1.6567	0.21616	0.46864
Methylmalonic acid	1.5363	0.2436	0.59013	33.58	2.74E-05	0.000137	1.2101	0.33805	0.56813
Glutamine	1.526	0.2461	0.59013	29.206	5.85E-05	0.000212	1.6534	0.21687	0.46864
Threonine	1.5233	0.24678	0.59013	37.708	1.42E-05	8.69E-05	2.049	0.14751	0.40565
Asparagine	1.3694	0.28788	0.63334	9.4504	0.007263	0.013315	1.2801	0.31497	0.56813
Maleic acid	1.2717	0.31767	0.672	35.584	1.98E-05	0.000109	1.2618	0.32085	0.56813
Proline	1.2223	0.3339	0.68018	6.6472	0.020215	0.033692	0.74227	0.54233	0.74961
Spermidine	1.0787	0.38616	0.72608	18.953	0.000493	0.001426	0.64772	0.59571	0.77898
8-Aminocaprylic acid	1.0356	0.4034	0.72608	12.557	0.002703	0.005718	0.82936	0.49696	0.71929
4-Aminobutyric acid	1.0214	0.40925	0.72608	14.22	0.001672	0.003832	1.2019	0.34088	0.56813
Aconitic acid	0.9442	0.44255	0.76064	6.2876	0.023319	0.037722	1.1576	0.35652	0.57672
Aspartic acid	0.82309	0.50011	0.80899	45.073	4.98E-06	3.42E-05	0.22334	0.87877	0.92947
Ethanolamine	0.58969	0.6306	0.91024	9.7506	0.006562	0.012445	1.8047	0.18688	0.4469
Alpha-ketoglutaric acid	0.56171	0.64798	0.91024	56.355	1.25E-06	9.85E-06	0.53348	0.66588	0.81679
Succinic acid	0.52103	0.67388	0.91024	29.77	5.28E-05	0.000207	1.6319	0.22154	0.46864
Fructose	0.51433	0.67822	0.91024	17.539	0.000696	0.001823	1.9354	0.16458	0.41176
5-Methoxytryptamine	0.45862	0.71498	0.91024	77.08	1.63E-07	2.00E-06	0.3833	0.76645	0.87688

Xylose	0.34647	0.79217	0.91024	11.255	0.004026	0.007909	0.67828	0.57799	0.77535
Ribitol	0.34127	0.79582	0.91024	95.924	3.67E-08	6.72E-07	1.3387	0.29691	0.56813
Glycine	0.31074	0.81732	0.91024	31.561	3.85E-05	0.000163	0.26312	0.85093	0.92947
Allose	0.29835	0.82608	0.91024	74.342	2.07E-07	2.00E-06	2.8212	0.072119	0.30512
Chlorogenic acid	0.29655	0.82734	0.91024	73.739	2.19E-07	2.00E-06	1.0577	0.39449	0.61991
D-(glycerol 1-phosphate)	0.26289	0.85109	0.91024	17.226	0.000753	0.001882	0.95766	0.43656	0.64895
Sucrose	0.24937	0.86059	0.91024	15.896	0.001061	0.002536	2.0974	0.14084	0.40565
Threitol/erythritol	0.17044	0.91478	0.9493	17.89	0.000638	0.001754	0.4751	0.70398	0.84171
Caffeic acid	0.11276	0.95137	0.96898	6.7168	0.019671	0.033692	1.3293	0.29974	0.56813
Citric acid	0.027802	0.99349	0.99349	32.028	3.55E-05	0.000163	0.04832	0.98541	0.98541

Table S20 Two-way repeated measures (within subjects) ANOVA of metabolites affected by PVY strain, time and their interaction during local and systemic infection of Russet Burbank

	Strain(F.val)	Strain(raw.p)	Strain(adj.p)	Time(F.val)	Time(raw.p)	Time(adj.p)	Interaction(F.val)	Interaction(raw.p)	Interaction(adj.p)
Myo-inositol	26.327	2.00E-06	5.49E-05	19.518	0.000431	0.001129	11.15	0.000338	0.006205
L-Allothreonine	22.791	5.09E-06	9.33E-05	17.14	0.000769	0.001839	23.235	4.50E-06	0.000247
Beta-Alanine	5.3672	0.00948	0.111	44.359	5.48E-06	2.77E-05	7.5415	0.0023	0.026131
Dehydroascorbic acid	5.0958	0.01152	0.111	28.443	6.73E-05	0.000247	0.66735	0.58427	0.75194
Phenylalanine	5.0275	0.012109	0.111	6.3053	0.023154	0.036386	12.635	0.000173	0.004745
Lysine	2.4294	0.10304	0.45613	7.1826	0.01643	0.027821	1.6525	0.21706	0.49744
D-Glyceric acid	2.2252	0.12475	0.45613	13.71	0.001931	0.003934	0.26842	0.8472	0.91365
Sucrose	2.1577	0.13298	0.45613	53.992	1.64E-06	1.29E-05	3.7741	0.031915	0.15957
Threitol/erythritol	2.1372	0.1356	0.45613	7.141	0.016693	0.027821	1.6934	0.20847	0.49744
Fructose	2.1021	0.14021	0.45613	103.42	2.17E-08	5.98E-07	7.3872	0.002524	0.026131
5-Methoxytryptamine	2.0963	0.14099	0.45613	12.244	0.002969	0.005631	5.6469	0.00779	0.053558
Maleic acid	1.9444	0.16316	0.48604	18.786	0.000513	0.001282	0.28277	0.83708	0.91365
Proline	1.9147	0.1679	0.48604	27.409	8.17E-05	0.000281	4.8208	0.014103	0.077566
Phosphate	1.6755	0.21218	0.52268	40.068	1.00E-05	4.41E-05	3.2327	0.050267	0.20874
Ribitol	1.6455	0.21858	0.52268	49.421	2.84E-06	1.74E-05	0.64543	0.59706	0.75194
Glutamine	1.5764	0.23406	0.53639	6.3832	0.022444	0.036306	2.0168	0.15215	0.38038
Succinic acid	1.4816	0.25726	0.54776	16.575	0.000889	0.001955	1.3382	0.29707	0.55202

Putrescine	1.4751	0.25894	0.54776	32.311	3.39E-05	0.000133	7.1873	0.002851	0.026131
Chlorogenic acid	1.3382	0.29706	0.57511	44.284	5.53E-06	2.77E-05	1.2842	0.31368	0.55202
Citric acid	1.2841	0.3137	0.57511	61.8	6.95E-07	7.65E-06	1.2855	0.31326	0.55202
Serine	1.0515	0.39698	0.68641	10.47	0.005175	0.009487	3.057	0.058545	0.21467
Tryptophan	0.97418	0.42932	0.69449	7.3521	0.015405	0.027332	6.3999	0.004694	0.036879
Alpha-ketoglutaric acid	0.7392	0.54399	0.74799	19.729	0.00041	0.001128	0.63781	0.60156	0.75194
Threonine	0.55153	0.6544	0.82898	16.584	0.000887	0.001955	0.71039	0.55985	0.75194
8-Aminocaprylic acid	0.54553	0.65819	0.82898	25.847	0.000111	0.000338	0.15797	0.923	0.97011
Asparagine	0.51036	0.6808	0.83034	26.506	9.71E-05	0.000314	1.2608	0.32117	0.55202
Fucose	0.48948	0.69447	0.83034	39.785	1.04E-05	4.41E-05	0.67107	0.58213	0.75194
Allose	0.4369	0.72964	0.84225	81.648	1.10E-07	1.52E-06	2.4443	0.10163	0.29419
Aspartic acid	0.40656	0.75036	0.84225	95.45	3.79E-08	6.96E-07	1.2767	0.31605	0.55202
Ethanolamine	0.37113	0.77491	0.8524	49.94	2.67E-06	1.74E-05	0.91578	0.45546	0.69568
Mannose	0.34394	0.79394	0.85621	58.232	1.02E-06	9.33E-06	2.3558	0.11035	0.30347
Xylose	0.29978	0.82507	0.87267	110.3	1.38E-08	5.98E-07	2.9029	0.067056	0.23051
Glucose-1-phosphate	0.22255	0.87931	0.91249	14.966	0.001361	0.002879	0.079669	0.97011	0.97011
Glycine	0.19023	0.9015	0.91819	13.378	0.002125	0.004173	0.91032	0.45798	0.69568
Oxoproline	0.11658	0.94908	0.94908	20.83	0.000319	0.000922	0.56169	0.648	0.76467

Table S21 Two-way between subjects ANOVA of metabolites affected by PVY strain, cultivar and their interactionat 7dpi. Color legend: Blue: metabolites affected by strain; Grey: metabolites affected by cultivar; Yellow: metabolites affected by strain-cultivar interaction; Adjusted *p*-value cutoff of 0.05 was used.

Metabolite	Strain(F.val)	Strain(adj.p)	Cultivar(F.val)	Cultivar(adj.p)
L-Allothreonine	46.035	2.32E-06	28.334	0.000741
Myo-inositol	35.422	7.29E-06	0.01779	0.93
Quinic acid	17.35	0.000499	0.84632	0.54184
Phenylalanine	13.768	0.001443	6.13	0.070626
Dehydroascorbic acid	11.315	0.003381	4.4723	0.11361
Ribitol	8.8963	0.009552	26.046	0.000957
Sucrose	7.6514	0.01639	0.7417	0.55639
Allose	7.451	0.01639	4.5841	0.11272
Maleic acid	7.1389	0.017622	0.54607	0.61986
Fumaric acid	6.7628	0.020067	0.24787	0.71849
Putrescine	6.1565	0.02705	0.39055	0.66374
Tryptophan	5.6774	0.034323	22.014	0.001889
Threonic acid	4.9315	0.053971	11.911	0.012808
Phosphate	4.4341	0.068045	38.354	0.000232
D-Glyceric acid	3.1104	0.14369	9.1026	0.027618
5-Methoxytryptamine	2.5308	0.18768	17.516	0.004199
Caffeic acid	2.3117	0.2142	100.24	1.46E-06
Spermidine	2.0799	0.25537	11.876	0.012808
Chlorogenic acid	1.7614	0.31907	11.112	0.015164
D-(glycerol 1-phosphate)	1.3477	0.38751	15.613	0.005613
Alpha-ketoglutaric acid	1.2335	0.42447	7.9664	0.038941
Xylose	0.97144	0.54064	16.09	0.005442
Aspartic acid	0.90227	0.54554	7.4395	0.044717
Fructose-6-phosphate	0.89586	0.54554	35.819	0.000257
Glucose-6-phosphate	0.62166	0.68278	18.846	0.003412
Methylmalonic acid	0.54102	0.71395	13.107	0.010341
8-Aminocaprylic acid	0.47695	0.74409	61.458	1.95E-05
